# Anti-myeloma activity of the CXCR4 antagonist WZ811

**DOI:** 10.1007/s00109-026-02650-4

**Published:** 2026-02-17

**Authors:** Danka Cholujova, Nikoleta Csicsatkova, Zuzana Valuskova, Katarina Suroviakova, Maria Elisabeth Marinkovicova, Anna Strizova, Jan Sedlak, Jana Jakubikova

**Affiliations:** 1 Cancer Research Institute, Department of Tumor Immunology, Biomedical Research Center, V. V. I, Slovak Academy of Sciences, Dubravska Cesta 9, 84505 Bratislava, Slovakia; 2 Centre for Advanced Materials Application, Slovak Academy of Sciences, Dubravska Cesta 9, 84511 Bratislava, Slovakia

**Keywords:** Multiple myeloma, CXCR4, Myeloma stem cell-like side population, Tumor microenvironment

## Abstract

**Abstract:**

The CXCR4-CXCL12 axis is crucial for the interaction between malignant plasma cells (PC) and their microenvironment in multiple myeloma (MM). Here, we show that CXCR4 expression is upregulated in MM cell lines and PCs during both premalignant and active stages of MM, compared to normal PCs from healthy donors. The CXCR4 antagonist WZ811 reduced the viability of MM PCs and cell lines, while tumor microenvironment cells from both MM patients and healthy donors exhibited significant resistance. In vivo, WZ811 significantly reduced tumor burden and improved survival. WZ811-mediated MM cell death involved disruption of mitochondrial transmembrane potential, externalization of transmembrane phosphatidylserine, activation of caspases, increased levels of autophagic proteins, and an increase in G_0_/G_1_ phase of the cell cycle. WZ811 also eliminated the MM stem cell-like side population, though slight resistance was observed with stromal cells. Additionally, WZ811 increased levels of CXCL12 and extracellular matrix molecules collagen IV and laminin in MM cells. Combining WZ811 with anti-MM agents showed synergism with doxorubicin, dexamethasone, bortezomib, lenalidomide, and pomalidomide, while antagonism was observed with carfilzomib, supporting the clinical assessment of WZ811 in MM.

**Key messages:**

- WZ811 reduced the viability of myeloma primary cells and cell lines.
- WZ811 reduced tumor burden and improved survival in vivo xenograft model.
- WZ811 mechanisms involved apoptotic and autophagic cell death.
- WZ811 eliminated the MM stem cell-like side population.
- WZ811 synergized with DOX, DEX, BTZ, LEN, and POM.

**Supplementary Information:**

The online version contains supplementary material available at 10.1007/s00109-026-02650-4.

## Introduction

Multiple myeloma (MM) constitutes a neoplastic proliferation of clonal plasma cells within the bone marrow microenvironment, characterized by monoclonal protein secretion and end-organ damage per CRAB criteria, with bone pain (73% prevalence), anemia, renal failure, and hypercalcemia predominant. MM arises through sequential genetic instability, initiating with primary cytogenetic hits such as hyperdiploidy or IgH translocations (e.g., t(4;14), t(11;14), t(14;16)), compounded by secondary aberrations including gain(1q), del(17p), MYC dysregulation, or RAS mutations that foster proliferation and therapy resistance. In addition, bone marrow stromal interactions and tumor microenvironment remodeling drive subclonal evolution, exacerbating disease heterogeneity [1, 2]. MM treatment integrates risk-adapted induction regimens, autologous stem cell transplantation (ASCT), and maintenance therapy to optimize progression-free survival (PFS) and minimal residual disease (MRD) negativity; ASCT, following high-dose chemotherapy, reinfuses patient-derived hematopoietic stem cells to rescue hematopoiesis, yielding superior median PFS (59.4 versus 27.5 months in non-complete responders) and extending overall survival, particularly in high-risk cytogenetics, with tandem ASCT further enhancing complete response rates by 25% and reducing progression risk by 30%. However, cell mobilization failure in 20–30% of patients, exacerbated by prior lenalidomide exposure, age, tumor burden, and MM microenvironmental CXCR4 overexpression on progenitors and tumor cells, delays ASCT and worsens outcomes, as standard G-CSF protocols fail despite CXCR4 antagonists like plerixafor or motixafortide [3–5]. Novel CXCR4 inhibitors are thus essential to overcome poor mobilization, disrupt stromal protection, and potentiate ASCT efficacy in MM.

The CXCL12/CXCR4 axis critically drives MM pathogenesis by mediating tumor cell homing, retention, survival, proliferation, and chemoresistance within the bone marrow microenvironment, with CXCL12 secreted by stromal cells and endothelium binding CXCR4 on MM cells to promote adhesion, migration into protective niches, and resistance to bortezomib. CXCR4/CXCL12 interactions activate key signaling cascades, including PLC/PKC, PI3K/Akt, MAPK/ERK, JAK/STAT, and NF-κB, that sustain MM growth, while also influencing Wnt/β-catenin, SHH-GLI-Nanog, and Notch pathways to enhance stemness and microenvironmental crosstalk via cytokines (IL-6, VEGF) and matrix metalloproteinases (MMPs). Aberrant CXCR4 expression further promotes osteoclastogenesis, angiogenesis, EMT (via Twist/Snail), and BM egress under hypoxia [6–8], positioning it as a prognostic marker and therapeutic target whose inhibition disrupts stroma-tumor interactions, mobilizes cells, induces apoptosis, and sensitizes to chemotherapy. Clinically approved CXCR4 antagonists, including plerixafor (AMD3100/Mozobil; FDA-approved 2008) and motixafortide (BL-8040; FDA-approved 2023), enhance hematopoietic stem/progenitor cell (HSPC) mobilization in MM when combined with G-CSF, with plerixafor additionally demonstrating chemosensitization to bortezomib despite lacking direct anti-tumor approval [3, 9]. WZ811, a potent small-molecule CXCR4 antagonist (EC50 0.3–1.2 nM), distinguishes itself through non-chelating chemistry, mitigating plerixafor-associated cardiotoxicity risks, while potently inhibiting CXCL12-mediated cAMP modulation, proliferation, migration, and PI3K/AKT/mTOR signaling in hematologic malignancies, including leukemia xenografts [10–13]. Unlike mobilization-centric agents, WZ811 exhibits direct anti-tumor potential by suppressing tumor growth and enhancing chemotherapy sensitivity (e.g., docetaxel), positioning it as a promising candidate for MM therapy.

In our study, we found upregulated CXCR4 expression in MM cell lines and malignant PCs during the stages of MM, compared to healthy donors (HD). The CXCR4 antagonist WZ811 reduced the viability of primary MM patient-derived PCs and MM cell lines, with significant resistance observed in tumor microenvironment cells derived from both MM patients and HD. Moreover, WZ811 significantly decreased tumor burden, leading to a notable survival benefit. The cellular and molecular mechanisms of WZ811 involved apoptotic and autophagic cell death, accumulation in the G_0_/G_1_ phase of the cell cycle, elimination of the MM stem cell-like side population, and upregulation of CXCL12 and ECM molecules in MM cells. To enhance the clinical response rate, combining WZ811 with anti-MM agents demonstrates synergism with DOX, DEX, BTZ, LEN, and POM, while antagonism is observed with CFZ as well as varied antagonistic-additive-synergistic effects are noted with MEL in MM cell lines, providing a basis for further clinical investigation of WZ811 in MM.

## Materials and methods

### Reagents

The CXCR4 antagonist WZ811, bortezomib (BTZ; Velcade), lenalidomide (LEN; CC5013), and pomalidomide (POM; CC4043) were procured from Selleck Chemicals (Houston, TX, USA). In addition, doxorubicin (DOX), dexamethasone (DEX), and melphalan (MEL) were sourced from Sigma-Aldrich (St. Louis, MO, USA).

### Primary cells and cell lines

MM cell lines MM.1S (RRID:CVCL_8792) and U266 (RRID:CVCL_0566) were sourced from the American Type Culture Collection (ATCC, Manassas, VA), while OPM-2 (RRID:CVCL_1625) and L-363 (RRID:CVCL_1357) cell lines were acquired from the Deutsche Sammlung von Mikroorganismen und Zellkulturen GmbH (DSMZ, Braunschweig, Germany). The chemosensitive cell line RPMI 8226-S (RRID:CVCL_0014) and its sublines resistant to doxorubicin RPMI-Dox6 (RRID:CVCL_J432) and RPMI-Dox40 (RRID:CVCL_J431), mitoxantrone RPMI-MR20 (RRID:CVCL_0509), and melphalan RPMI-LR5 (RRID:CVCL_J434) were generously provided by Dr. William S. Dalton (Lee Moffitt Cancer Center, Tampa, FL, USA). Human MM cell lines OPM-1 (RRID:CVCL_5210), KMS-11 (RRID:CVCL_2989), OCI-My5 (RRID:CVCL_E332), OCI-My7 (RRID:CVCL_E333), and JJN-3 (RRID:CVCL_2078) were kindly donated by Dr. Teru Hideshima (Dana Farber Cancer Institute, Boston, MA, USA). The human bone marrow stromal cell line HS-5 (RRID:CVCL_3720) was obtained from the ATCC. All MM cell lines were cultured in RPMI 1640 medium (Cellgro, Mediatech, VA) supplemented with 10% heat-inactivated fetal bovine serum (FBS; Harlan, Indianapolis, IN), 100 U/ml penicillin, 100 μg/ml streptomycin, and 2 mM L-glutamine (GIBCO, Grand Island, NY) at 37 °C in a 5% CO2 atmosphere. The HS-5 stromal cell line was cultured in Dulbecco’s modified Eagle medium (DMEM; Cellgro, Mediatech, VA) supplemented with 10% heat-inactivated fetal bovine serum (FBS; Harlan, Indianapolis, IN), 100 U/ml penicillin, 100 μg/ml streptomycin, and 2 mM L-glutamine (GIBCO, Grand Island, NY) at 37 °C in 5% CO_2_. Cell lines were authenticated by cell profiling (Promega PowerPlex Fusion System kit; Promega, Madison, WI) in the past 3 years. The presence of mycoplasma was periodically tested by PCR.

Fresh mononuclear cells (MNCs) were isolated from patients and healthy volunteers using Ficoll-Hypaque (Pharmacia, Piscataway, NJ, USA) density sedimentation. Patient MM cells were purified by cell sorting with a CD138-PE monoclonal antibody to isolate CD138+ plasma cells (MM cells) and non-plasma tumor microenvironment (accessory) cells from freshly obtained bone marrow samples. Cells were cultured in RPMI 1640 medium containing 20% heat-inactivated FBS, 100 U/ml penicillin, 100 μg/ml streptomycin, and 2 mM L-glutamine, and maintained at 37 °C in a 5% CO2 atmosphere. This study received approval from the Biomedical Research Center Institutional Review Board under the protocol Myelom 001. Informed consent was obtained from all patients and healthy volunteers in accordance with the Declaration of Helsinki protocol.

#### Evaluation of WZ811 sensitivity in cell-based assays

The sensitivity of MM cells to WZ811 was assessed using cell viability assays, including the colorimetric MTT assay and the luminescent CellTiter-Glo (CTG) assay. Apoptosis quantification was conducted via the annexin V-fluorescein isothiocyanate assay by flow cytometry. Mitochondrial membrane potential was evaluated by flow cytometry using the JC-1 fluorescent probe. Cell cycle alterations in DNA content of nuclei stained with propidium iodide (PI) were analyzed by flow cytometry. Cell division in MM cells was assessed using carboxyfluorescein diacetate succinimidyl ester (CFSE) staining, which also allowed differentiation between CFSE-labeled MM cells and unlabeled HS-5 stromal cells. The proportion of non-viable cells was determined through PI staining. Immunofluorescence analysis of primary antibodies directed at CXCL12, laminin, and collagen IV, followed by incubation with allophycocyanin (APC) fluorescence-tagged secondary antibody, was performed on both MM cells labeled with CFSE alone or in coculture with unlabeled HS-5 stromal cells. Comprehensive details of these methodologies are provided in the Supplementary Methods.

#### Molecular profiling analyses and functional assays of WZ811 treatment

The molecular sequelae of CXCR4 by WZ811 treatment in MM cells were evaluated using quantitative real-time PCR (qRT-PCR) following RNA extraction and cDNA synthesis, as detailed in the Supplementary Methods. Protein-level signaling modifications were assessed via western immunoblotting, also described in the Supplementary Methods and Supplementary pdf file. The functional impact of WZ811 on the stem cell-like side population (SP) was assessed using the Hoechst 33342 assay via flow cytometry. MM cells were labeled with carboxyfluorescein diacetate succinimidyl ester (CFSE), and non-viable cells were identified using 7-aminoactinomycin D (7-AAD) staining, both alone and in a co-culture model with unlabeled BMSC HS-5 cells, as detailed in the Supplementary Methods.

### Statistical analysis

The statistical significance of differences between WZ811-treated and control samples was determined using one-way ANOVA followed by Dunnett’s multiple comparison test. Significance levels were denoted as *p < 0.05, **p < 0.01, ***p < 0.001, and ****p < 0.0001. In vivo overall survival was analyzed using the Kaplan-Meier log-rank test. Data are expressed as mean ± standard deviation (SD). The half maximal effective concentration (EC_50_) of WZ811 was calculated using CalcuSyn software (Biosoft, Ferguson, MO, USA).

## Results

### CXCR4 expression levels in MM cells

**Fig. 1:**
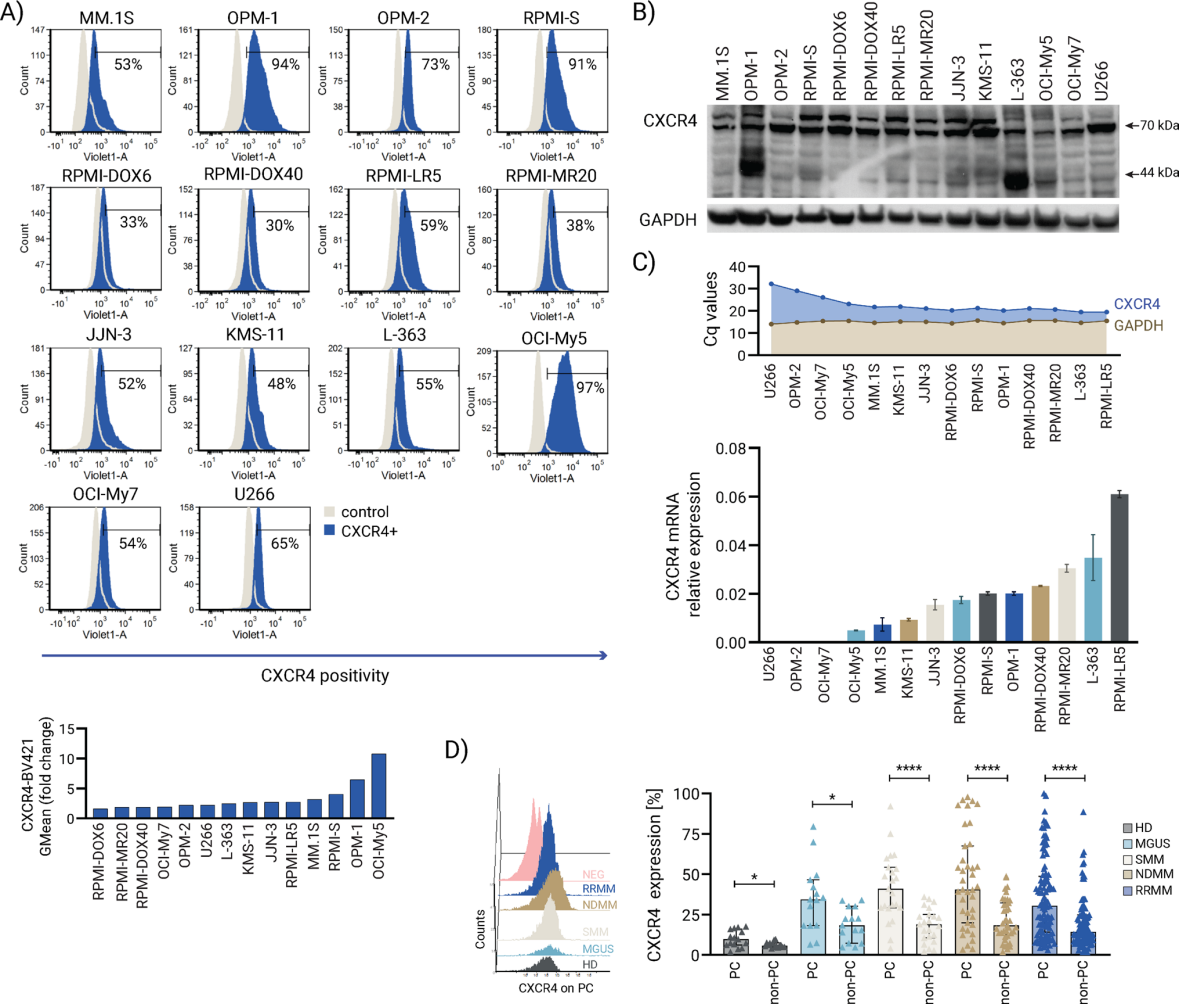
CXCR4 expression levels in MM. **A** The expression of CXCR4 at the protein level was analyzed in a panel of 14 MM cell lines (MM.1S, OPM-1, OPM-2, RPMI-S, RPMI-DOX6, RPMI-DOX40, RPMI-LR5, RPMI-MR20, JJN-3, KMS-11, L-363, OCI-My5, OCI-My7, and U266) by flow cytometry (FACS Canto II flow cytometer, Becton Dickinson), represented by histograms of CXCR4-BV421 positive expression (blue) compared to the negative Ig isotype control (grey), and **B** by western immunoblot analysis using anti-CXCR4 and anti-GAPDH (used as a loading control) antibodies. The fold change of the geometric mean of CXCR4 expression relative to the isotype fluorescent control is shown. The data are representative from three independent experiments. **C** CXCR4 expression at the transcriptional mRNA level was examined using RT-PCR, showing mRNA levels of CXCR4 in MM cell lines. The data are from three independent experiments and are presented as means ± standard deviation. **D** The representative 3D flow cytometry-based histograms depict CXCR4 expression on plasma cells (PC) in healthy donors (HD; black), monoclonal gammopathy of unknown significance (MGUS; light blue), smoldering MM (SMM; grey), newly diagnosed MM (NDMM; brown), and relapsed/refractory MM (RRMM; blue), compared to negative Ig isotype control (NEG; pink). The column bar graph shows the percentage of CXCR4 expression on normal PC (expressing CD138+/CD45+/CD38+) and non-PC of healthy donors (HD; *n* = 10), and malignant PC (expressing CD138+/CD45low/CD38++) and non-PC from primary bone marrow-derived cells of premalignant MGUS (*n* = 15) and SMM (*n* = 23), as well as active NDMM (*n* = 39) and RMM (*n* = 102) MM patients analyzed by flow cytometry and calculated using the Mann-Whitney U test, *p < 0.05 and ****p < 0.0001

The CXCR4-CXCL12 axis plays an important role in MM-microenvironment interaction in association with the growth and survival of malignant plasma cells involved in the evolution and progression of MM disease [8]. To assess the role of CXCR4 in MM, we evaluated the expression levels of CXCR4 in a panel of 14 MM cell lines (MM.1S, OPM-1, OPM-2, RPMI-S, RPMI-DOX6, RPMI-DOX40, RPMI-LR5, RPMI-MR20, JJN-3, KMS-11, L-363, OCI-My5, OCI-My7, and U266 cells) by flow cytometry. We observed a population of 30 to 97% of MM cells expressing CXCR4, with the strongest >90% CXCR4 expression in RPMI-S, OPM-1, and OCI-My5 cells (Fig. 1A). In addition, a pronounced increase in the expression of CXCR4 in MM cell lines was revealed using western immunoblotting analysis, with the highest CXCR4 expression observed in OPM-1 cells (Fig. 1B). Similarly, elevated mRNA levels of CXCR4 were also detected in MM cells using real-time RT-PCR, indicating the overexpression of CXCR4 in MM cell lines at the transcriptional level (Fig. 1C). Higher mRNA levels of CXCR4 were observed in RPMI-MR20, L-363, and RPMI-LR5 cells compared to other MM cell lines. Upregulated CXCR4 expression levels were assessed in primary malignant plasma cells (PC) from premalignant (MGUS; *n* = 15) and smoldering MM (SMM; *n* = 23), as well as active (newly diagnosed MM (NDMM; *n* = 39) and relapsed/refractory MM (RRMM; *n* = 102)) MM patients and compared to normal PC from healthy donors (HD; *n* = 10), with tumor cells exhibiting profoundly higher percentage of CXCR4-expressing cells compared to normal plasma cells (*p* =  <0.0001). Similarly, non-PC within the MM microenvironment of all MM patient stages versus non-PC of HD demonstrated a significantly elevated percentage of CXCR4 (*p* = 0.0056). Furthermore, the disparities in CXCR4 expression between PC and non-PC of respective stages increased in more advanced MM stages, including SMM, NDMM, and RRMM (Fig. 1D). The high activity of CXCR4 may be especially necessary for MM cell growth and function; therefore, a reduction in CXCR4 is crucially needed.

### WZ811 decreases MM cell viability

**Fig. 2:**
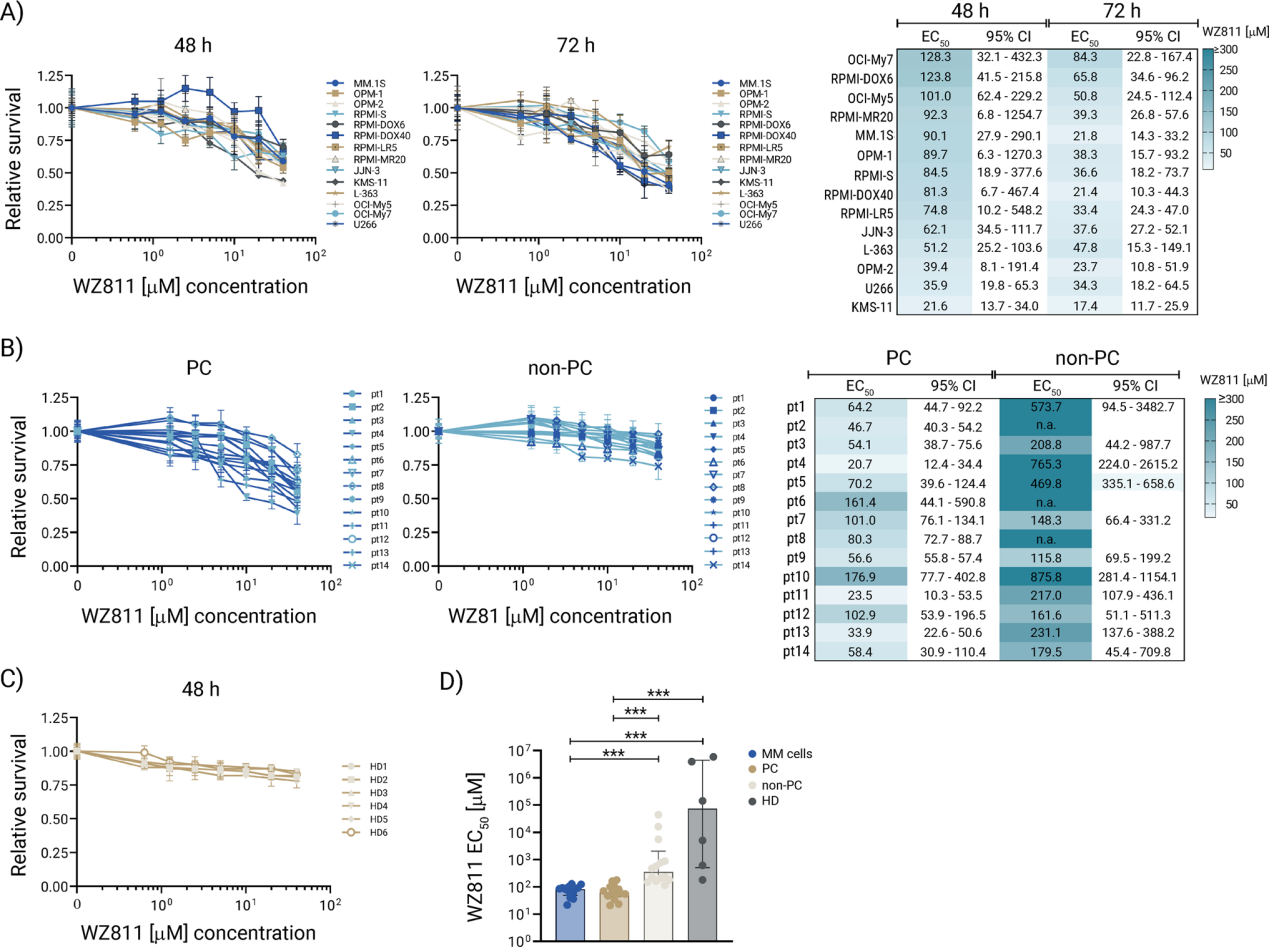
WZ811 decreases survival of MM cells. **A** MM cell lines (MM.1S, OPM-1, OPM-2, RPMI-S, RPMI-DOX6, RPMI-DOX40, RPMI-LR5, RPMI-MR20, JJN-3, KMS-11, L-363, OCI-My5, OCI-My7, and U266 cells) were exposed to indicated concentrations (0.625, 1.25, 2.5, 5, 10, 20, and 40 μM) of WZ811 for 48 h and 72 h. The cell survival was determined by MTT assay, and the EC_50_ values of WZ811 were examined in MM cell lines for 48 h and 72 h using the CalcuSyn software. **B** Freshly sorted malignant bone marrow PC (expressing CD138+/CD45low/CD38++; *n* = 14) and tumor microenvironment cells (non-PCs; *n* = 14), both obtained from the same primary MM patient samples, were exposed to the indicated concentrations (1.25, 2.5, 5, 10, 20, and 40 μM) of WZ811 for 48 h. The cell survival was assessed by CellTiterGlo assay, and the EC_50_ values of WZ811 in both PC and non-PC populations were determined for 48 h using the CalcuSyn software. If an exceptionally high EC_50_ value was observed, it was reported as “n.a.” **C** Freshly isolated peripheral blood mononuclear cells (MNCs) from healthy donors (HD; *n* = 6) were exposed to indicated concentrations (0.625, 1.25, 2.5, 5, 10, 20, and 40 μM) of WZ811 for 48 h, and cell survival was assessed by MTT assay. The EC_50_ values of WZ811 were determined in HD for 48 h using the CalcuSyn software. EC50 data are not presented due to exceptionally high values. Each treatment with a specific concentration of WZ811 was performed in triplicate. The presented data are mean ± standard deviation, expressed as survival/viability relative to untreated controls. EC_50_ (half maximal effective concentration) is shown with lower and upper 95% confidence intervals (CI). **D** The median EC_50_ values of WZ811 in MM cell lines, malignant PC, and non-PC of primary cells derived from MM patients, as well as MNCs of HD, were compared and calculated using One-way ANOVA on ranks followed by Kruskal-Wallis multiple comparison test, with ***p < 0.001

To examine whether CXCR4 antagonist WZ811 has an anti-proliferative effect on MM cells, we incubated 14 MM cell lines with different concentrations (0–40 μmol/l) of WZ811 for 24 h, 48 h, and 72 h, and determined viability using the MTT assay. Cell survival decreased in a concentration-dependent manner in response to WZ811 at 24 h (Supplementary Figure S1) and 48 h (Fig. 2A). The concentration-dependent reduction in cell viability was determined by EC_50_ values (the concentration of 50% reduced cell survival) for WZ811 using CalcuSyn software. In a time-dependent manner, EC_50_ concentrations dropped on average 1 to 2-fold from 24 to 48 h in most MM cell lines (Supplementary Figure S1 and Fig. 2A). The highest anti-proliferative effect of WZ811 was observed at 72 h in the growth of all MM cell lines tested, including those resistant to conventional therapeutic agents (such as RPMI-DOX40, RPMI-LR-5, and RPMI-MR20) (Fig. 2A). Viability was uniformly inhibited by WZ811, with EC_50_ values ranging from 17 to 84 μM (including outliers OCI-My7 and RPMI-DOX6 cells) at 72 h. The majority of MM cell lines exposed to WZ811, with calculated EC_50_ values of 20–40 μM including MM.1S, RPMI-S and OPM1 cells, resulted in a 2-fold decrease in the number of viable cells following 48 h of culture.

We next evaluated the impact of WZ811 on primary malignant PC and non-PC of the tumor microenvironment, both derived from the BM of different MM patients (*n* = 14, Fig. 2B; Supplementary Table 1 lists the clinical characteristics of the primary MM samples used in the study) compared to PBMNCs from HD (*n* = 6) at 48 h (Fig. 2C). The significant anti-MM activity of WZ811 was confirmed on malignant PC derived from primary MM patients exposed to WZ811, with EC_50_ values ranging from 21 to 177 μM, compared to non-PC, which resulted in an average 66-fold increase in EC_50_ values. In addition, healthy PBMNCs after exposure to WZ811 were significantly more resistant than MM tumor cells, whether MM cell lines or primary patient-derived PC cells. Similarly, non-PC of the tumor microenvironment from MM patients showed significant resistance to WZ811 compared to both PC and MM cell lines (Fig. 2D). Of note, the sensitivity of MM cells, either primary MM patient-derived PC or MM cell lines, to WZ811 was significantly higher than that of non-malignant surrounding/accessory MNCs (depleted of malignant PC) derived from MM patients and HD.

### WZ811 induces apoptotic and autophagic cell deaths in MM cells

**Fig. 3:**
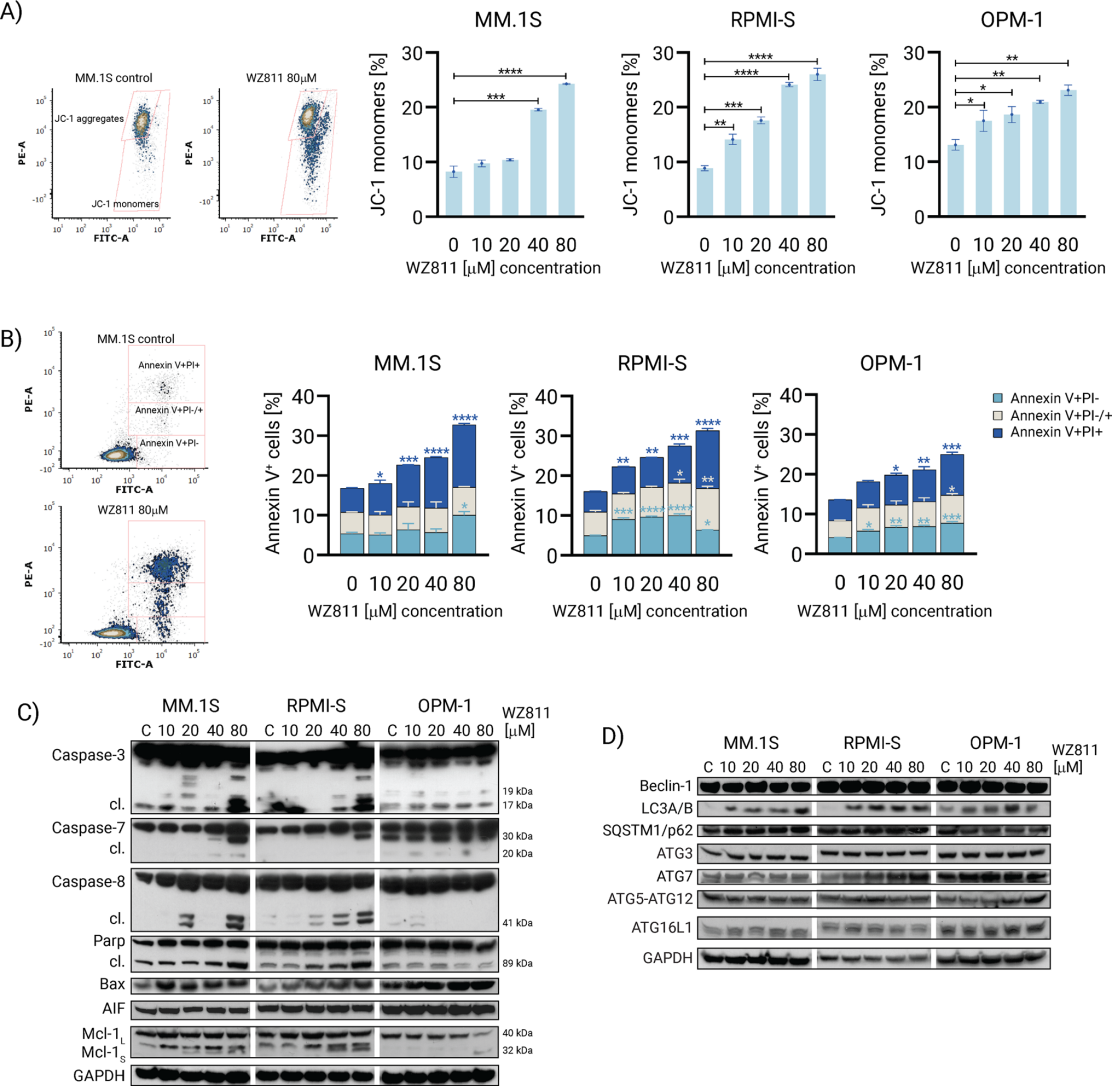
WZ811 induces apoptosis and autophagy in MM cells. MM.1S, RPMI-S, and OPM-1 cells were treated with indicated concentrations (10, 20, 40, and 80 μM) of WZ811 for 72 h. **A** Representative flow cytometry-based dot plots showing the proportions of JC-1 aggregates versus JC-1 monomers in WZ811-treated MM.1S cells at 80 μM compared to control cells for 72 h. Decrease of mitochondrial membrane potential in MM cells after exposure to WZ811 was examined by staining with the fluorescent JC1 dye, indicating increased levels of JC-1 monomers, and analyzed by a FACS Canto II flow cytometer. **B** Representative flow cytometry-based dot plots showing the proportions of early apoptotic (Annexin V+/PI−), late apoptotic (Annexin V+/PI+/−), and necrotic (Annexin V+/PI+) cells in WZ811-treated MM.1S cells at 80 μM compared to control cells for 72 h. Induction of Annexin V+/Pi−, Annexin V+/Pi+/−, and Annexin V+/Pi+ cells was determined with Annexin V-FITC and Pi staining and analyzed by a FACS Canto II flow cytometer. The data are from three independent experiments and presented as means ± standard deviation. Significant differences between treatments and control were identified by one-way ANOVA followed by Dunnett’s multiple comparison test with *p < 0.05, **p < 0.01, ***p < 0.001, and ****p < 0.0001. Modulation of **C** apoptotic- and **D** autophagic-related regulation proteins was evaluated in MM cells after exposure to WZ811 and compared to control (DMSO-treated) cells by Western blot analysis. Western blot analyses were performed on whole cell lysates (20 μg of proteins/lane) using anti-caspase-3, -caspase-7, -caspase-8, -PARP, -Bax, -AIF, -Mcl-1, -Beclin-1, -LC3A/B, -SQSTM1/p62, -ATG3, -ATG7, -ATG5-12, -ATG16L1, and -GAPDH (serving as a loading control) antibodies. Data are representative of two independent experiments

To delineate the mechanism of WZ811-mediated MM cell death, we initially examined the disruption of mitochondrial transmembrane potential (Δψm) in WZ811-treated MM cell lines (MM.1S, RPMI-S, and OPM1), one of the earliest hallmarks of apoptosis, assessed by flow cytometry using JC-1 fluorescent dye. The aggregation of JC-1 dimers directly correlates with mitochondrial membrane potential (MMP), and the breakdown of these dimers in dying cells leads to the production of JC-1 monomers, indicative of the loss of MMP. We observed a significant but slight decrease in MMP in WZ811-treated MM cells in a dose-dependent manner, as evidenced by an increase in JC-1 monomers at 72 h (Fig. 3A), with the trend being most pronounced in RPMI-S cells followed by OPM-1 and MM.1S cells, consistent with changes observed in MMP at 24 h (Supplementary Figure S2A).

Next, apoptosis was confirmed using the gold standard apoptotic hallmark Annexin V and PI staining. An early apoptotic event, associated with the externalization of transmembrane phosphatidylserine, is detected by Annexin V staining, while late necrotic events internalize PI into the nucleus of dead cells. In addition, late apoptosis events are indicated by intermediate staining of both fluorochromes. Treatment of MM cell lines (MM.1S, RPMI-S, and OPM1) with WZ811 revealed a moderate induction of apoptosis, as evidenced by an increased percentage of Annexin V+/PI− cells, characteristic of the early apoptotic population detected even at lower concentrations of WZ811 (10 μM) in RPMI-S and OPM1 cells, followed by an increase in the percentage of necrosis demonstrated by Annexin V+/PI+ cells in all three MM cell lines at 24 h (Supplementary Figure S2B) and 72 h (Fig. 3B). Furthermore, Annexin V+/PI± cells, characteristic of late apoptotic events, were predominantly observed at higher concentrations of WZ811 (40 μM and 80 μM).

Western immunoblotting analyses demonstrated the activation of caspase-3, caspase-7, caspase-8, and cleavage of PARP protein primarily by higher concentrations of 20, 40, and 80 μM WZ811 in MM1.S and RPMI-S cells. In addition, a notable upregulation of the pro-apoptotic Bcl-2 family regulator Bax and Mcl-1S isoform was predominantly observed in WZ811-treated MM.1S and RPMI-S cells. Conversely, no discernible changes were detected in the levels of pro-apoptotic AIF in response to WZ811 treatment (Fig. 3C and Supplementary Figure S3). Furthermore, a more detailed analysis of autophagic downstream targets revealed elevated levels of LC3A/B, SQSTM1/p62 (only MM.1S cells), ATG7 (MM.1S and RPMI-S cells), and ATG16L1 (only OPM-1 cells) proteins, with no significant alterations in Beclin-1, ATG3, and ATG5-ATG12 proteins (Fig. 3D and Supplementary Figure S3), suggesting that the anti-MM activity of WZ811 is not solely reliant on apoptosis but also involves autophagy. In summary, these data suggest that the anti-MM activity of WZ811 is moderately associated with both apoptosis and autophagy induction.

### WZ811 induces cell cycle arrest in MM cells

**Fig. 4:**
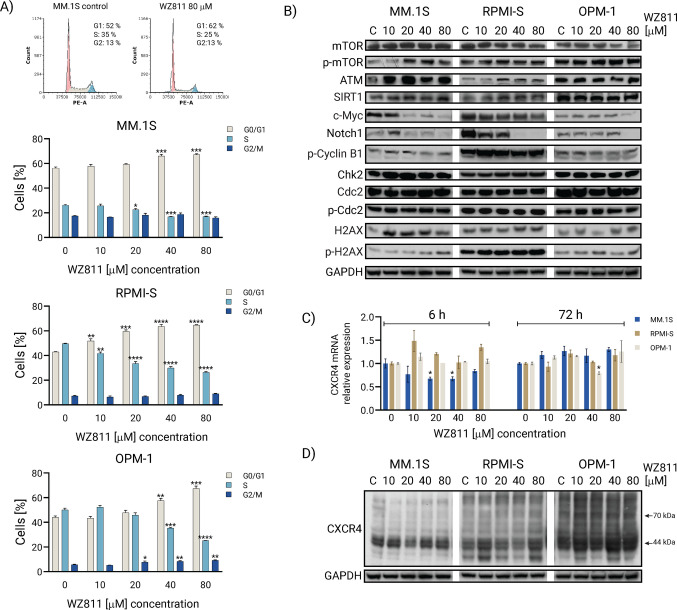
WZ811 induces G0/G1 cell cycle arrest in MM cells. **A** Flow cytometry-based cell cycle analysis of control and WZ811-treated MM.1S cells at 80 μM for 72 h with the distribution of cells in G0/G1, S, and G2/M phase showing the representative data out of three experiments. MM.1S, RPMI-S, and OPM-1 cells were exposed to indicated concentrations (10, 20, 40, and 80 μM) of WZ811 for 72 h, and their cell cycle profiles were analyzed using propidium iodide (Pi) staining. The distribution of cells in G0/G1, S, and G2/M phase was measured by a FACS Canto II flow cytometer and analyzed with De Novo FCS Express software. The data are from three independent experiments and are presented as means ± standard deviation. Significant differences between treatments and control were identified by one-way ANOVA followed by Dunnett’s multiple comparison test with *p < 0.05, **p < 0.01, ***p < 0.001, and ****p < 0.0001. **B** MM cell lines (MM.1S, RPMI-S, and OPM-1 cells) treated with WZ811 (10, 20, 40, and 80 μM) for 72 h were examined for changes in the levels of cell cycle regulatory and signaling molecules by western blot analysis. Western blot analyses of whole cell lysates (20 μg of proteins per lane) were immunoblotted using anti-mTOR, -p-mTOR, -ATM, -SIRT1, -c-Myc, -Notch1, -p-Cyclin B1, -Chk2, -Cdc2, -p-Cdc2, -histone H2AX (H2AX), -p-histone H2AX (p-H2AX), and -GAPDH (used as a loading control) antibodies. Results are representative of two independent experiments. **C** CXCR4 expression at the transcriptional mRNA level was examined after treatment with WZ811 (10, 20, 40, and 80 μM) for 6 h and 72 h in MM cell lines (MM.1S, RPMI-S, and OPM-1 cells) using RT-PCR analysis. Data are from three independent experiments and are presented as relative CXCR4 expression normalized to control ± standard deviation. **D** MM cell lines (MM.1S, RPMI-S, and OPM-1 cells) treated with WZ811 (10, 20, 40, and 80 μM) for 6 h were examined for changes in the expression levels of CXCR4 by western blot analysis. Western blot analyses of whole cell lysates (20 μg of proteins per lane) were immunoblotted using anti-CXCR4 and -GAPDH (used as a loading control) antibodies. Results are representative of two independent experiments

To evaluate the cell cycle profile of WZ811-treated MM cell lines, we analyzed the distribution of cell cycle phases in MM.1S, RPMI-S, and OPM1 cell lines following exposure to increasing concentrations of WZ811 (10–80 μM) for 24 and 72 h. Treatment of MM.1S and OPM-1 cells with WZ811 slightly increased the percentage of cells in the G_2_/M and S phases at 24 h, respectively (Supplementary Figure S4). Conversely, a significant increase in the G_0_/G_1_ phase accompanied by a reduction in the S phase was observed in MM.1S, RPMI-S, and OPM1 cells after 72 h of exposure to WZ811 in a concentration-dependent manner (Fig. 4A).

To evaluate the signaling modulation triggered by WZ811, we determined the activation of the mTOR signaling pathway (only MM.1S cells), the increase of SIRT1 (only RPMI-S cells), and the decrease of c-Myc and Notch1 proteins in MM cell lines after WZ811 treatment using immunoblotting analysis. Moreover, western blot analysis of WZ811-induced changes in cell cycle regulators revealed upregulation of ATM, Chk2, H2AX, and p-H2AX molecules, while no changes were observed in the expression of p-Cyclin B1, Cdc2, and p-Cdc2 proteins after 72 h of treatment (Fig. 4B and Supplementary Figure S5).

Interestingly, WZ811 did not decrease mRNA CXCR4 expression levels by more than 0.5-fold at concentrations of 10–80 μM or in a time-dependent manner (6 and 72 h), as determined by RT-PCR (Fig. 4C). Additionally, WZ811 did not alter the expression of CXCR4 at the protein level in MM cell lines at 6 h (Fig. 4D) or at later time points (data not shown). Notably, WZ811 triggered a significant increase in the G_0_/G_1_ phase of the cell cycle in MM cells, along with modulations in cell cycle signaling regulators such as ATM, Chk2, H2AX, and p-H2AX, as well as other signaling molecules, including p-mTOR, SIRT1, c-Myc, and Notch1.

### WZ811 decreases the stem cell-like side population fraction and increases CXCL12 and extracellular matrix proteins collagen IV and laminin in MM cells

**Fig. 5:**
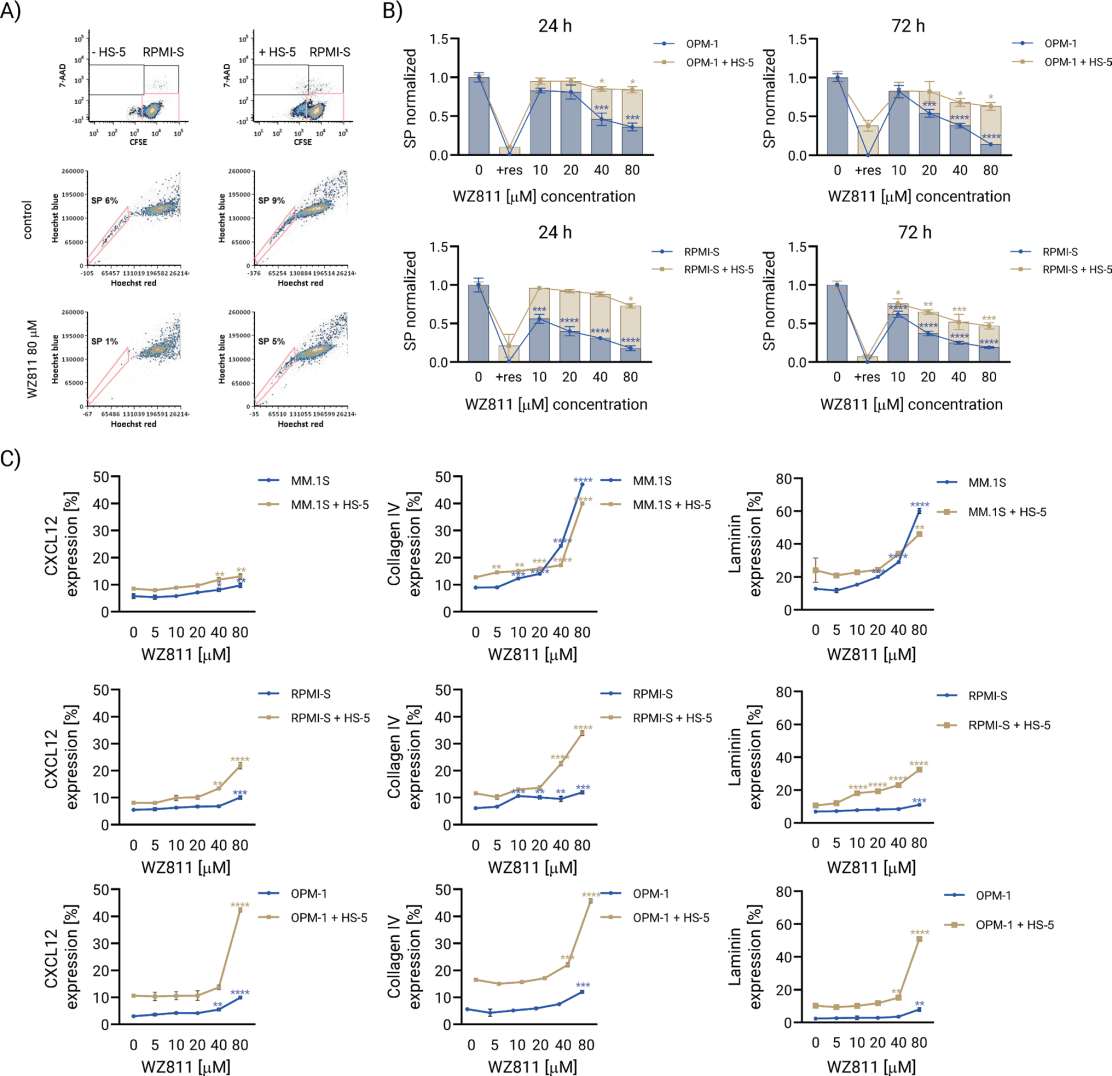
WZ811 decreases the side population fractions and increases the expression extracellular matrix proteins CXCL12, collagen IV and laminin in MM cells. **A** RPMI-S cells were stained with carboxyfluorescein diacetate succinimidyl ester (CFSE) and cultured either alone or in the presence of BMSC HS-5 cells. Non-viable MM (CFSE+/7-AAD+) and HS-5 stromal (CFSE-/7-AAD+) cells were identified by 7-aminoactinomycin D (7-AAD) staining (upper row). Side population (SP) cells were determined by low intracellular staining with Hoechst 33342 fluorescence dye, gating only on CFSE+/7-AAD− RPMI-S cells, either alone or in the presence of BMSC HS-5 cells (middle row). The SP cell fraction was examined after treatment with 80 μM WZ811 for 24 h in RPMI-S cells, either alone or in the presence of BMSC HS-5 cells (lower row), using a FACS Aria Special Sorter UV laser flow cytometer (Becton Dickinson Biosciences, San Jose, CA, USA). **B** OPM-1 and RPMI-S cells, stained with CFSE, either alone or in the presence of BMSC HS-5 cells, were exposed to the indicated concentrations (10, 20, 40, and 80 μM) of WZ811 for 24 and 72 h. The normalized proportion of the SP cell fraction was calculated relative to the control (untreated) MM cells, both alone and in co-culture with BMSC HS-5 cells. **C** The expression of CXCL12, collagen IV, and laminin at the protein level in MM.1S, RPMI-S, and OPM-1 cells, stained with CFSE and treated with different concentrations of WZ811 (5, 10, 20, 40, and 80 μM) for 72 h, either alone or in the presence of unstained BMSC HS-5 cells, was analyzed by flow cytometry. The data are from three independent experiments performed in triplicate and are presented as means ± standard deviation. Significant differences between treatments and control were identified by one-way ANOVA followed by Dunnett's multiple comparison test, with *p < 0.05, **p < 0.01, ***p < 0.001, and ****p < 0.0001

The cancer stem cell contributes to disease recurrence and, similar to bone marrow stromal cells in the tumor microenvironment, confers drug resistance, representing a major obstacle in cancer treatment. Therefore, we evaluated the activity of WZ811 on MM stem cell-like SP cells in MM cells (with a high proportion of SP fraction, such as RPMI-S and OPM-1 cells) both alone and in the presence of HS-5 bone marrow stromal cells (BMSCs). MM cells (RPMI-S and OPM-1) were stained with carboxyfluorescein diacetate succinimidyl ester (CFSE) dye and then treated with increasing doses of WZ811 for 24 and 72 h, either alone or in the presence of unstained HS-5 BMSCs (Fig. 5A, upper row). Gating on CFSE+/7-AAD− MM cells, SP cells were identified by low intracellular content of Hoechst 33342 in MM cells versus MM cells treated with WZ811, as determined by flow cytometry analysis (Fig. 5A, middle and lower rows). This SP fraction disappeared when cells were treated with reserpine (data not shown). Treatment with WZ811 at concentrations of 40 μM and 80 μM for 24 h, and even from 20 μM for 72 h, significantly decreased the proportion of MM stem cell-like SP cells in OPM-1 cells, as well as at concentrations of 10–80 μM in RPMI-S cells. In the context of BMSCs, the anti-SP activity of WZ811 was slightly attenuated in both MM cell lines. However, despite higher resistance at 24 h, the SP fraction decreased more significantly in RPMI-S cells treated with 10–80 μM of WZ811 compared to OPM-1 cells treated with 40–80 μM of WZ811 at 72 h (Fig. 5B).

MM (MM.1S, RPMI-S, and OPM-1) cells were stained with CFSE and cultured either alone or in the presence of BMSCs HS-5 cells. Next, we evaluated the changes in the expression of CXCL12 and ECM proteins collagen IV and laminin in MM cells at the protein level, either alone or in the presence of HS-5 cells, triggered by WZ811 using flow cytometry. WZ811 treatment significantly upregulated the expression of CXCL12 and ECM molecules collagen IV and laminin in both MM cells alone and in the context of BMSCs. Interestingly, the pattern of increased CXCL12 and ECM molecules was similar in MM.1S cells alone and in the presence of BMSCs (Fig. 5C). However, in RPMI-S and OPM-1 cells (with a high proportion of SP fraction), CXCL12 and both ECM molecules were more upregulated in the context of BMSCs. In summary, WZ811 eliminated the SP fraction and increased the levels of CXCL12 and ECM molecules collagen IV and laminin in MM cells. In the context of the BMSCs of the tumor microenvironment, the anti-SP activity of WZ811 was only slightly decreased and was associated with a more pronounced upregulation of CXCL12 and ECM molecules collagen IV and laminin in MM cells.

### WZ811 suppresses MM burden in a xenograft mouse model *in vivo*

**Fig. 6:**
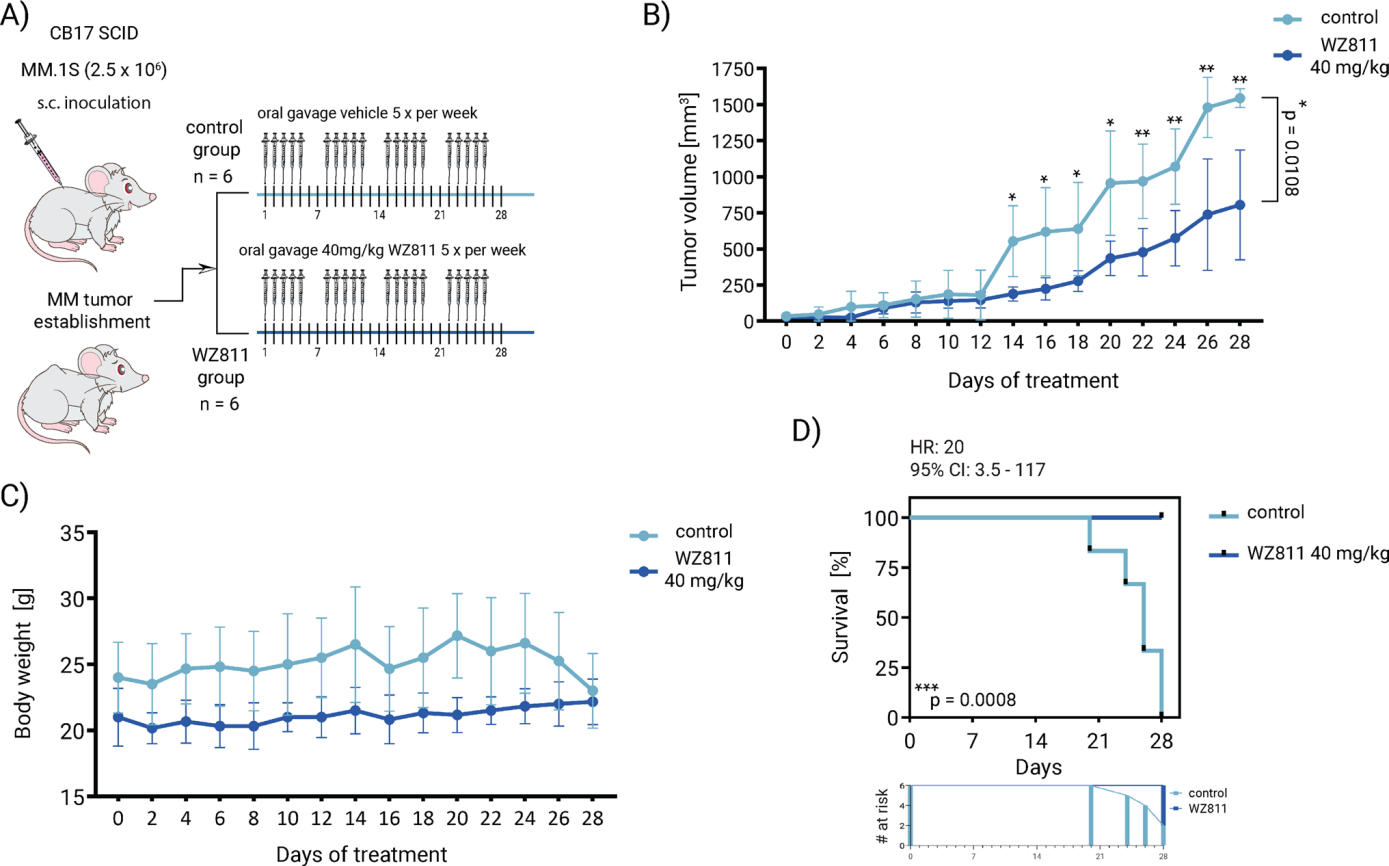
WZ811 decreases tumor burden in a mouse xenograft model of human MM in vivo. **A** MM.1S cells (2.5 × 10^6^ cells in 200 μl PBS) were injected subcutaneously into CB17/SCID mice, and treatment was initiated when tumors became palpable. Tumor-bearing mice were randomly assigned into two groups (six mice per group) treated with WZ811 (diluted in water for injection with 10% DMSO and 5% Tween 80) at a concentration 40 mg/kg or vehicle control (water with 10% DMSO and 5% Tween 80), both administered by oral gavage (once a day for 5 consecutive days followed by 2 days off, and repeated for 5 weeks) in the xenograft MM mouse model. Tumor diameters were measured every 2–3 days with a caliper, and tumor volumes were calculated according to the formula of the volume of an ellipse: *V* = 4/3π × (*a*/2) × (*b*/2)^2^, where *a *and *b* correspond to the longest and shortest tumor diameter, respectively. Representative photograph shows vehicle-treated tumors (upper row) versus WZ811-treated tumors (lower row) at the end of experiment using Pro 12MP camera system. **B** The mice treated with WZ811 showed significant suppression of tumor growth compared to the mice treated with vehicle. Significant differences in the volume of the tumors between WZ811- and vehicle-treated mice were evaluated by two-way ANOVA followed by Tukey multiple comparison test with *p = 0.0108. The data are presented as the means ± standard deviation. **C** Body weights of mice were measured every 2–3 days and compared between WZ811-treated and vehicle-treated mice. **D** Kaplan-Meier analysis of overall survival showed a significant increase in the survival of WZ811-treated mice compared to vehicle-treated control mice, calculated by Kaplan-Meier log-rank test, with ***p = 0.0008. Numbers at risk (# at risk), hazard ratio (HR), and 95% confidence interval (CI) are shown

To determine whether WZ811 is effective in eliminating MM cells in vivo, we used a xenograft myeloma model established after s.c. injection of CB17/SCID mice with MM.1S cells. Tumor-bearing mice were randomly assigned into two groups to receive either 40 mg/kg of WZ811 (*n* = 6) or vehicle control (*n* = 6), both administered by oral gavage (once a day for 5 consecutive days followed by 2 days off, and repeated for 4 weeks) in the xenograft MM mouse model (Fig. 6A). WZ811 significantly reduced tumor volumes, with a significantly decreased tumor burden observed from day 14 of treatment in WZ811-treated mice cohort compared to vehicle-treated control mice until the end of the experiment (*p* = 0.0108; Fig. 6B). Importantly, the treatment was well tolerated, with no significant body weight changes between WZ811- and vehicle-treated mice groups (Fig. 6C). WZ811 tumor growth inhibition also resulted in a significant survival benefit at the end of the study, on day 28, with WZ811 monotherapy group living significantly longer (*p = *0.0008, WZ811- versus vehicle-treated control mice; Fig. 6D). Taken together, these results show that WZ811 reduces MM tumor burden and improves survival as monotherapy in a xenograft MM mouse model.

### WZ811 in combination with anti-MM agents increases anti-MM cytotoxicity *in vitro*

**Fig. 7:**
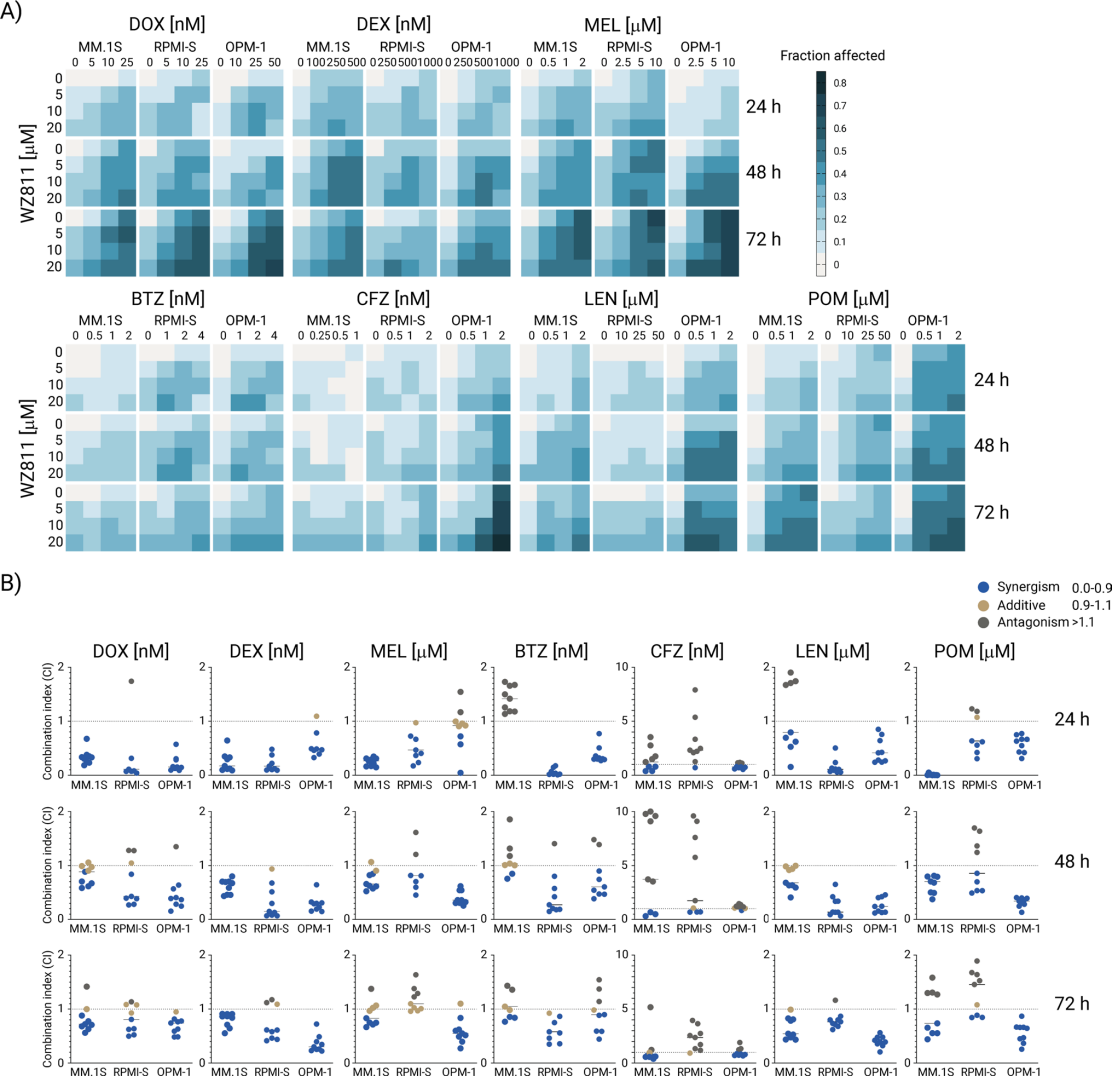
WZ811 in combination with anti-MM agents increases anti-MM activity in vitro. MM.1S, RPMI-S, and OPM-1 cells were exposed to the indicated concentrations (5, 10, and 20 μM) of WZ811 in combination with conventional agents: doxorubicin (DOX), dexamethasone (DEX) and melphalan (MEL), as well as novel agents: bortezomib (velcade; BTZ), carfilzomib (CFZ), lenalidomide (LEN), and pomalidomide (POM) anti-MM drugs for 24 h, 48 h and 72 h. Cell survival was then assessed by MTT assay, and combination effects were determined by the CalcuSyn software. **A** Fractions-affected (Fa; the ratio of the number of nonviable MM cells to the total number of MM cells) were visualized in a color-coded format and compared to treatment with each drug alone. **B** Isobologram analysis was performed to calculate the combination index (CI) for each combination by the Chou–Talalay method. The x-axis corresponds to the fractional effect at various combination doses in MM cells, and the y-axis represents the CI; CI < 1 indicates drug synergy, CI = 1 is considered additive, and CI > 1 indicates antagonism. All experiments were performed in triplicate. Data represent the median with interquartile range (IQR) of triplicate cultures

We next investigated combinations of WZ811 with conventional (dexamethasone, doxorubicin, and melphalan) and novel (bortezomib, carfilzomib, lenalidomide, and pomalidomide) anti-MM therapies used in standard clinical care of MM patients. The combined anti-tumor activity of WZ811 with these agents was evaluated in MM cell lines (MM.1S, RPMI-S, and OPM-1 cells) at 24, 48, and 72 h using the MTT assay. Combination effects were analyzed using CalcuSyn software, showing the fraction affected (Fa) in heatmaps (Fig. 7A) and combination indices (CI) for each combination in MM cells in isobologram graphs compared to single treatments (Fig. 7B).

Conventional anti-MM agents such as doxorubicin (DOX), dexamethasone (DEX), and melphalan (MEL) exhibited strong synergistic effects (as defined by criteria detailed by Chou-Talalay [14]) when combined with WZ811 in MM cell lines, with CI < 1 for the majority of conditions after 24 h of treatment, except for the antagonistic-additive effects of MEL in OPM-1 cells. Prolonged treatment for 48 and 72 h enhanced cell killing with DOX or DEX and WZ811, except for some drug doses that showed diminished synergistic effects when combined with WZ811. Conversely, combinations of WZ811 with MEL had either additive or antagonistic effects in MM.1S and RPMI-S cells at 72 h, whereas synergism was observed only in OPM-1 cells.

The first-generation proteasome inhibitor bortezomib (BTZ) in combination with WZ811 showed synergism in RPMI-S and OPM-1 cells, except for MM.1S cells, which predominantly exhibited antagonistic effects at 24 h, shifting to additive and slight synergism at 48 and 72 h. Conversely, the second-generation proteasome inhibitor carfilzomib (CFZ), with WZ811, exhibited mostly antagonistic effects in MM.1S and RPMI-S cells at 24 and 48 h, with synergism observed at 72 h in MM.1S and OPM-1 cells at all time points. In combination of WZ811 with the first-generation immunomodulatory (IMiD) agent lenalidomide (LEN), significant synergism was detected in all three MM cell lines, except for a few antagonistic doses in MM.1S cells at 24 h, which shifted to additive to synergistic effects at 48 and 72 h. Similarly, synergistic effects were observed with the combination of WZ811 and the second-generation IMiD pomalidomide (POM), although this combination transitioned towards antagonism at 72 h, mostly in RPMI-S cells. Notably, the combination of WZ811 with conventional anti-MM agents shows synergism with DOX and DEX and varied effects with MEL in MM cell lines, while the combination of WZ811 with proteasome inhibitors and IMIDs shows synergism with BTZ, LEN, and POM, and antagonism is noted with CFZ, highlighting differential responses across different MM cell lines and time points.

## Discussion

Approximately 60% of primary MM cells in the BM express CXCR4, with its expression inversely correlated with disease activity [15]. Previously, we revealed that high levels of CXCR4 on PC subclones and plasmablasts are associated with prolonged overall survival (OS) and progression-free survival (PFS), while low levels of CXCR4 on PC subclones are linked to inferior PFS and OS outcomes [16]. Contrary to findings suggesting an inverse correlation between CXCR4 expression and disease activity, MM cells exhibit high levels of CXCR4 in peripheral blood. However, in the BM, CXCR4 expression diminishes in response to elevated CXCL12 levels, leading to receptor internalization within MM cells. This internalization into the cytoplasm by CXCL12 signifies poor prognosis, while nuclear localization of CXCR4 indicates a better prognosis [17]. Furthermore, lower methylation frequency of CXCR4 genes in MM patients is linked to improved progression-free survival [18]. Elevated serum levels of CXCL12 are associated with increased osteolytic disease [19]. In our study, we observed a significantly elevated percentage of CXCR4-expressing cells in primary malignant PC of MM patients across premalignant MGUS, SMM, and active MM stages, compared to normal PC from healthy donors. Additionally, we determined CXCR4 overexpression in MM cell lines at both mRNA and protein levels, with the highest expression (> 90%) observed in RPMI-S, OPM-1, and OCI-My5 cells. Surrounding non-PC of the MM microenvironment showed a significantly upregulated percentage of CXCR4-expressing cells compared to non-PC from healthy donors. Furthermore, the disparities in CXCR4 expression between PCs and non-PCs increased in more advanced MM stages, including SMM, NDMM, and RRMM stages. Considering MM’s pathogenesis, the CXCR4/CXCL12 axis emerges as both a prognostic marker and a therapeutic target. This insight fuels the development of novel anti-MM therapeutics, ranging from antagonists to natural or synthetic small molecules, peptides, and monoclonal antibodies.

Understanding the CXCR4/CXCL12 axis has driven the development of anti-CXCR4 therapies. Approved CXCR4 antagonists, such as plerixafor (AMD3100/Mozobil; FDA-approved 2008) and motixafortide (BL-8040; FDA-approved 2023), potently enhance hematopoietic stem/progenitor cell (HSPC) mobilization in MM when combined with G-CSF, achieving 18–20 × 10⁶ CD34 + cells/kg and 76.8-fold HSPC increases over controls, respectively, while plerixafor demonstrates bortezomib chemosensitization (48.5% response rate) despite lacking direct anti-tumor approval [3, 9, 20]. Clinical-stage agents like mavorixafor (X4P-002; orally bioavailable for WHIM syndrome with cancer promise), ulocuplumab (BMS-936565; phase I/II MM trials showing bortezomib synergy), and lilotomab (BKT140) exhibit safety and tumor mobilization but face limitations including hematological toxicities and discontinued development (e.g., F50067) [21–23]. Preclinical inhibitors, including AMD3465, panobinostat (LBH589; FDA-approved with anti-MM synergy), PF-06747143, olaptesed pegol, and antibodies like LY2624587 or MDX-1338, further disrupt CXCL12 binding to induce apoptosis, ADCC/CDC, or enable imaging-guided therapies (e.g., 68 Ga/177Lu-pentixafor); however, many have stalled due to toxicity or suboptimal pharmacokinetics [24–29]. Thus, the development of novel therapies targeting both direct anti-MM effects and spatial tumor heterogeneity, while inhibiting MM cell dissemination, is crucial.

A small-molecule antagonist of CXCR4, WZ811 binds both the ligand- and agonist-binding sites of the CXCR4 receptor, thereby blocking both the binding and signaling of SDF-1α [30]. Pharmacological evaluations reveal EC50 values of 0.3 nM for binding affinity, 1.2 nM for inhibition of CXCL2-mediated cAMP modulation, and 5.2 nM for suppression of SDF-1-induced Matrigel invasion [13, 31, 32]. In contrast, another study reported very weak anti-CXCR4 inhibitory activity of WZ811 [12]. Despite conflicting reports on its inhibitory activity, WZ811 demonstrates significant anti-tumorigenic potential in diffuse large B-cell lymphoma (DLBCL) and CLL by inhibiting cell proliferation and survival, inducing apoptosis in vitro, and blocking tumor growth in vivo [11, 33]. In our study, we observed the cytotoxic effects of WZ811 on MM cell lines, including those resistant to conventional anti-MM therapeutic agents, with determined EC_50_ values of 20–40 μM. The pronounced anti-MM activity of WZ811 was corroborated in malignant PC isolated from primary MM patients, exhibiting EC_50_ values ranging from 21 to 177 μM. The uniform, time- and dose-dependent antiproliferative effects (a twofold viability decrease at 20–40 μM in most lines, such as MM.1S/OPM-1; sustained activity in drug-resistant RPMI variants) confirm that cell line mutations do not bias CXCR4 inhibitor response, validating WZ811’s mechanism via CXCL12 blockade rather than mutation compensation. Primary PC sensitivity (despite patient-specific mutations), versus resistance in non-malignant surrounding/accessory cells, further supports this, as CXCR4 signaling protects malignant PCs in the BM niche without mutation dependency. This consistency across MM cell lines and primary cells strengthens WZ811’s therapeutic potential, bypassing conventional resistance pathways while selectively targeting MM over healthy cells. Furthermore, the reduced tumor burden caused by WZ811 was confirmed in vivo in an MM xenograft mouse model, with notably extended survival.

The extrinsic pathway activates caspase-8, which triggers effector caspases-3 and −7 to cleave substrates like PARP, causing apoptosis and cellular disassembly. In CLL, WZ811 induces apoptosis through the down-regulation of Bcl2, upregulation of Bax, and activation of caspase-3 [11]. In our study, WZ811 treatment induced apoptosis in MM cells, as confirmed by the externalization of transmembrane phosphatidylserine, and was associated with the activation of caspase-3, caspase-7, and caspase-8, as well as the cleavage of PARP protein. In addition, WZ811 triggered upregulation of the pro-apoptotic Bcl-2 family regulator Bax and depolarization of mitochondrial membrane potential, indicating involvement of the intrinsic mitochondrial apoptotic pathway. Mcl-1S, a pro-apoptotic isoform of the Mcl-1 gene, promotes Bax activity, inhibits Mcl-1L survival signals, and is linked to mitotic acceleration, DNA damage, and apoptosis [34]. The simultaneous activation of caspases (particularly −3, −7, and −8), cleavage of PARP, upregulation of Bax, and Mcl-1S expression suggests a well-coordinated apoptotic response driven by both intrinsic and extrinsic pathways.

Increased levels of CXCR4 led to higher cellular concentrations of key signaling proteins associated with autophagy, specifically the protein markers LC3 and beclin [35]. LC3, a key marker of autophagy, transitions from cytosolic LC3-I to lipidated LC3-II to associate with autophagosome membranes and interact with SQSTM1/p62, which regulates autophagy, targets ubiquitinated substrates, and activates the Nrf2 pathway for antioxidant responses and stress management. In LC3 lipidation, ATG7, an E1-like enzyme, facilitates the conjugation of LC3 to phosphatidylethanolamine (PE), while ATG16L1, as part of the ATG5-ATG12 complex, supports this process and aids in targeting ubiquitinated cargo for degradation [36]. Our study demonstrated that WZ8111 significantly increased the levels of LC3A/B, SQSTM1/p62, ATG7, and ATG16L1, highlighting an active autophagic process crucial for autophagosome formation, cellular homeostasis, and the degradation of damaged organelles and protein aggregates. Therefore, the interplay between autophagy and apoptosis appears to drive WZ811’s anti-MM activity.

ATM, a serine/threonine kinase activated by double-strand breaks (DSBs) and recruited by the MRN complex (MRE11-RAD50-NBS1), undergoes autophosphorylation at Ser1981 to initiate DNA damage response signaling. In response to DSBs, ATM phosphorylates H2AX at Ser139, converting it into γH2AX, which marks damage sites, recruits repair proteins, and signals DNA repair and checkpoint activation [37]. Chk2, activated by ATM phosphorylation at Thr68, mediates cell cycle arrest at G1/S and G2/M checkpoints, allowing time for DNA repair before cell division. Elevated p-H2AX levels indicate ongoing DNA repair, marking DSBs, recruiting repair proteins like BRCA1 and MDC1 [38]. Similar to a previous study in CLL [11], we observed a significant increase in the G_0_/G_1_ phase in MM cells after exposure to WZ811, along with upregulation of ATM, Chk2, H2AX, and p-H2AX, triggering DNA damage response pathways such as H2AX phosphorylation and Chk2-mediated checkpoint activation to prevent the propagation of damaged DNA.

Pharmacological inhibition of the CXCL12/CXCR4 axis with WZ811 also prevents CD41+ megakaryocyte migration in bleomycin-induced lung fibrosis and reduces T-cell migration [11, 39, 40]. In thyroid carcinoma and NSCLC, WZ811 suppresses tumor invasiveness, inhibits epithelial-mesenchymal transition (EMT), increases E-cadherin, and reduces Twist, vimentin, and Snail expression [41, 42]. In gastric cancer, WZ811 counteracts FER1L4-mediated suppression of CXCR4 and CXCL12, enhancing tumor growth and metastasis [43]. CXCR4 expression correlates positively with mTOR expression, predicting reduced PFS and OS in DLBCL patients co-expressing these markers [44]. The mTOR pathway, often activated in MM, drives tumor growth and survival by promoting protein synthesis and suppressing autophagy, contributing to therapy resistance. SIRT1 (Sirtuin 1), a NAD+-dependent deacetylase, inhibits mTOR signaling via protein deacetylation and enhances autophagy to eliminate damaged components, acting as a stress-response mechanism in MM [45]. In addition, WZ811 reduces osteotropism and activates the mTOR and p70-S6 cell signaling proteins in melanoma cells [35]. In our study, WZ811 treatment similarly resulted in the activation of the mTOR signaling pathway, an increase in SIRT1, and a decrease in c-Myc and Notch1 proteins in MM cells. Reduced c-Myc and Notch1 levels might further promote plasma cell differentiation, shift metabolism toward oxidative phosphorylation, and potentially modulate mTOR activity. Together, these changes suggest a shift toward differentiation and stress adaptation; however, due to the anti-myeloma activity of WZ811, they may also indicate a stress response in MM. Therefore, we hypothesize that targeting CXCR4 with WZ811 may constitute an effective therapeutic approach for MM.

MM plasma cells exhibit phenotypic plasticity, with a subset of MM stem-like cells influencing disease initiation and progression. In the proposed MM stem model, quiescent MM stem cells, serving as tumor initiators, display heightened expression of CXCR4, integrins, and adhesion molecules, facilitating MM cell motility, migration, and adherence to BMSCs. Conversely, proliferative MM stem cells, driving disease progression and the emergence of evolved subclones, exhibit minimal CXCR4 expression, suggesting its downregulation in advanced MM stages associated with relapse [46]. In our previous study, we identified stem-like SP cells expressing CD138 and a high level of ABCG2 transporter activity as an enriched source of tumor-initiating cells in MM. These SP cells demonstrated the ability to regenerate the original population with clonogenic and tumorigenic properties [47]. Besides HSCs, CXCR4 is expressed in SP cells in various cancer stem cells, including those in breast cancer, glioblastoma, and MM, and is linked to increased tumorigenicity, metastatic potential, and resistance to chemotherapy [47]. CXCR4 is also expressed in neural stem cells, mesenchymal stem cells, and cardiac progenitor cells [48]. This highlights the importance of CXCR4 expression in SP cells across various tissues and its role in mediating stem cell functions and therapeutic potential. Moreover, the anti-MM agent lenalidomide decreased the percentage and clonogenicity of SP cells, both in MM cells alone and in the context of BMSCs [47]. Treatment with WZ811 significantly reduced the proportion of stem cell-like SP cells in MM. In the context of BMSCs, although the anti-SP activity of WZ811 was attenuated in both MM cell lines at 24 h, the SP fraction decreased more significantly in RPMI-S cells compared to OPM-1 cells after 72 h of treatment. Our studies show that WZ811 effectively targets the SP fraction in MM cells, establishing a foundation for new therapeutic strategies aimed at eliminating subpopulations of MM cells, including potential stem cells.

CXCR4 is a key marker of bone metastasis in MM, universally upregulated in plasma cells. Its interaction with CXCL12 facilitates MM cell rolling on endothelium, recruitment of CXCL12-expressing stromal cells, and organ-specific homing, including to the liver, lungs, and bone marrow. This axis drives MMP synthesis, enabling ECM degradation, tumor cell detachment, bloodstream egression, and migration to metastatic sites [49]. We showed that WZ811 treatment triggered the upregulated expression of CXCL12 and ECM molecules, including collagen IV and laminin, in MM cells, and these effects were further enhanced in the presence of stromal cells. Treatment with WZ811 can alter signaling pathways within MM cells, potentially triggering feedback mechanisms that upregulate the expression of certain cytokines and chemokines, including CXCL12, either to maintain homeostasis or in response to stress induced by WZ811. Moreover, WZ811 may cause changes in stromal cells that indirectly promote the production of CXCL12 in MM cells. WZ811 may activate key signaling pathways that regulate ECM production, such as TGF-β, Wnt, or integrin signaling, leading to increased synthesis and deposition of ECM components, including collagen IV and laminin. By targeting SP cells or affecting cytokine production (such as CXCL12), WZ811 might modulate interactions between MM cells and stromal cells, leading to ECM remodeling and increased production of ECM proteins.

Combination drug therapy can target multiple pathways involved in cancer cell growth and survival by employing drugs with different mechanisms of action. Moreover, the likelihood of cancer cells simultaneously developing resistance to all agents is significantly reduced. Certain drug combinations exhibit synergistic interactions, where the combined therapeutic effect is greater than the sum of the individual effects, thus targeting different but complementary cellular pathways. The combination of the CXCR4 inhibitor WZ811 with conventional anti-MM agents demonstrates synergistic effects with DOX, an anthracycline that intercalates DNA, inhibits topoisomerase II, and generates free radicals leading to DNA damage and apoptosis, as well as with DEX, a corticosteroid that induces apoptosis. The combination of WZ811 with the alkylating agent MEL demonstrates a range of outcomes, from synergistic to additive to antagonistic, in various MM cell lines. The combination of WZ811 with proteasome inhibitors and IMIDs demonstrates synergistic effects with BTZ, LEN, and POM, while it exhibits antagonistic interactions with CFZ. Thalidomide further downregulates CXCR4 and CXCL12, while carfilzomib inhibits CXCR4 phosphorylation [50]. Moreover, WZ811 significantly enhances the sensitivity of CLL cells to docetaxel [11] and exhibits a synergistic effect in combination with the mTOR inhibitor everolimus in DLBCL cell lines [44]. For example, DOX and DEX may enhance the apoptotic pathways activated by WZ811, while MEL and CFZ may compete with or disrupt the mechanisms triggered by CXCR4 inhibition, depending on the cellular context. Furthermore, the interplay between WZ811’s inhibition of CXCR4 signaling and the proteasome/immune modulatory pathways targeted by BTZ, LEN, and POM may explain their synergistic effects. Using lower doses of each drug in combination therapy can reduce the incidence of dose-limiting toxicities associated with higher doses of single agents. For example, dexamethasone at lower doses reduces the risk of steroid-related side effects such as hyperglycemia and immunosuppression, doxorubicin at lower doses minimizes the risk of cardiotoxicity, and bortezomib at lower doses can reduce the risk of peripheral neuropathy. To maximize therapeutic outcomes, sequential or concurrent administration of drugs can be tailored to exploit specific vulnerabilities of cancer cells at different stages of the cell cycle or in distinct tumor microenvironments. Combination therapy can address tumor heterogeneity by targeting multiple subpopulations within the tumor, thereby reducing the likelihood of treatment failure due to clonal expansion of resistant cells, such as SP cells.

This study demonstrates the preclinical efficacy of WZ811 against MM through CXCR4 blockade, manifesting as apoptosis induction, autophagy promotion, cell cycle arrest, and modulation of MM-associated signaling pathways across diverse MM cell lines, ex vivo patient-derived plasma cells, and in vivo xenograft models. WZ811 further reduced the stem-like side population fraction and synergized with anti-MM agents, establishing a rationale for combination therapies to overcome stromal protection and resistance. However, reliance on transformed/immortalized cell lines and xenograft models introduces limitations, including incomplete recapitulation of human tumor heterogeneity, bone marrow microenvironment dynamics, and acquired resistance mechanisms prevalent in relapsed/refractory disease. Future investigations should advance WZ811 into Phase I clinical trials for relapsed/refractory MM, prioritizing pharmacokinetic/pharmacodynamic profiling, CXCR4/CXCL12 biomarker stratification, and safety assessments to mitigate potential cytopenias from prolonged blockade. This should be complemented by evaluation of rational combination strategies, multi-omics analyses of stress and survival pathways, and validation in humanized models to support durable anti-myeloma activity.

## Conclusions

In summary, we have demonstrated the anti-MM activity of the CXCR4 antagonist WZ811 across various MM cell lines in vitro, ex vivo isolated plasma cells, and in an MM xenograft mouse model in vivo, involving cellular and molecular mechanisms such as apoptosis induction, promotion of autophagy, cell cycle arrest, and modulation of several MM-associated signaling molecules. Furthermore, our preclinical in vitro study indicated that WZ811 significantly reduced the MM stem-like SP cell fraction and enhanced the therapeutic potential of several anti-MM agents, providing a framework for designing efficient therapeutic strategies utilizing WZ811 in combination therapies to combat MM.

## Supplementary Information

Below is the link to the electronic supplementary material.

ESM 1 (PNG 235 KB)

High Resolution Image (TIF 1.60 MB)

ESM 2 (PNG 198 KB)

High Resolution Image (TIF 1.96 MB)

ESM 3 (PNG 235 KB)

High Resolution Image (TIF 3.05 MB)

ESM 4 (PNG 198 KB)

High Resolution Image (TIF 1.15 MB)

ESM 5 (PNG 198 KB)

High Resolution Image (TIF 2.07 MB)

ESM 6 (DOCX 19.1 KB)

ESM 7 (DOCX 25.4 KB)

ESM 8 (PDF 14.5 MB)

ESM 9 (PDF 330 KB)

ESM 10 (XLSX 12.2 KB)

## Data Availability

All data generated and analyzed in this study are included in this published article and its Supplementary files.

## Abbreviations

MM: Multiple myeloma
BM: Bone marrow
BMSCs: Bone marrow stromal cells
BTZ: Bortezomib
CXCR4: Chemokine C-X-C motif receptor 4
DEX: Dexamethasone
DOX: Doxorubicin
ECM: Extracellular matrix
LEN: Lenalidomide
MEL: Melphalan
PC: Plasma cells
POM: Pomalidomide
SP: Side population
