# Supplementary figures and images for: Anti-myeloma activity of the CXCR4 antagonist WZ811
Source: J Mol Med (Berl). 2026 Feb 17;104(1):45. doi: 10.1007/s00109-026-02650-4 (PMC12913330; doi:10.1007/s00109-026-02650-4)

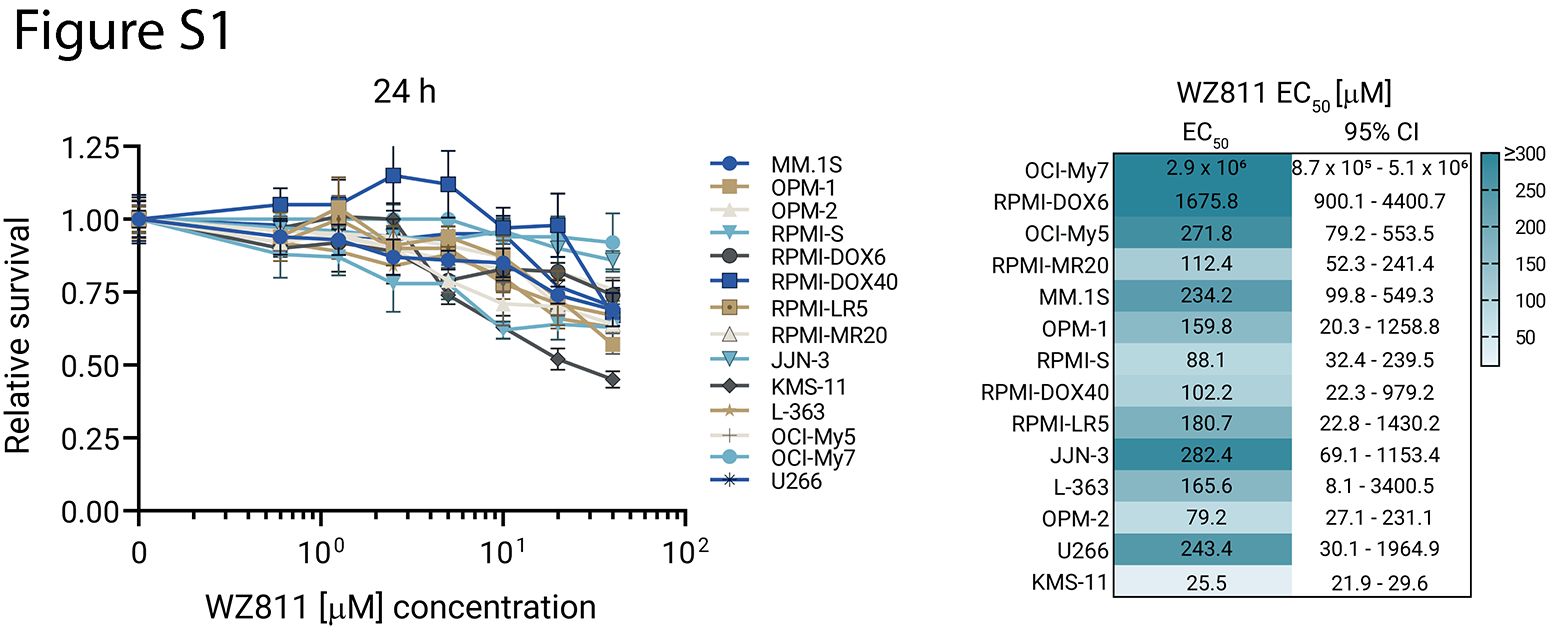

Supplement: Supplementary file 1 — (PNG 235 KB) [file 109_2026_2650_Fig8_ESM.png]

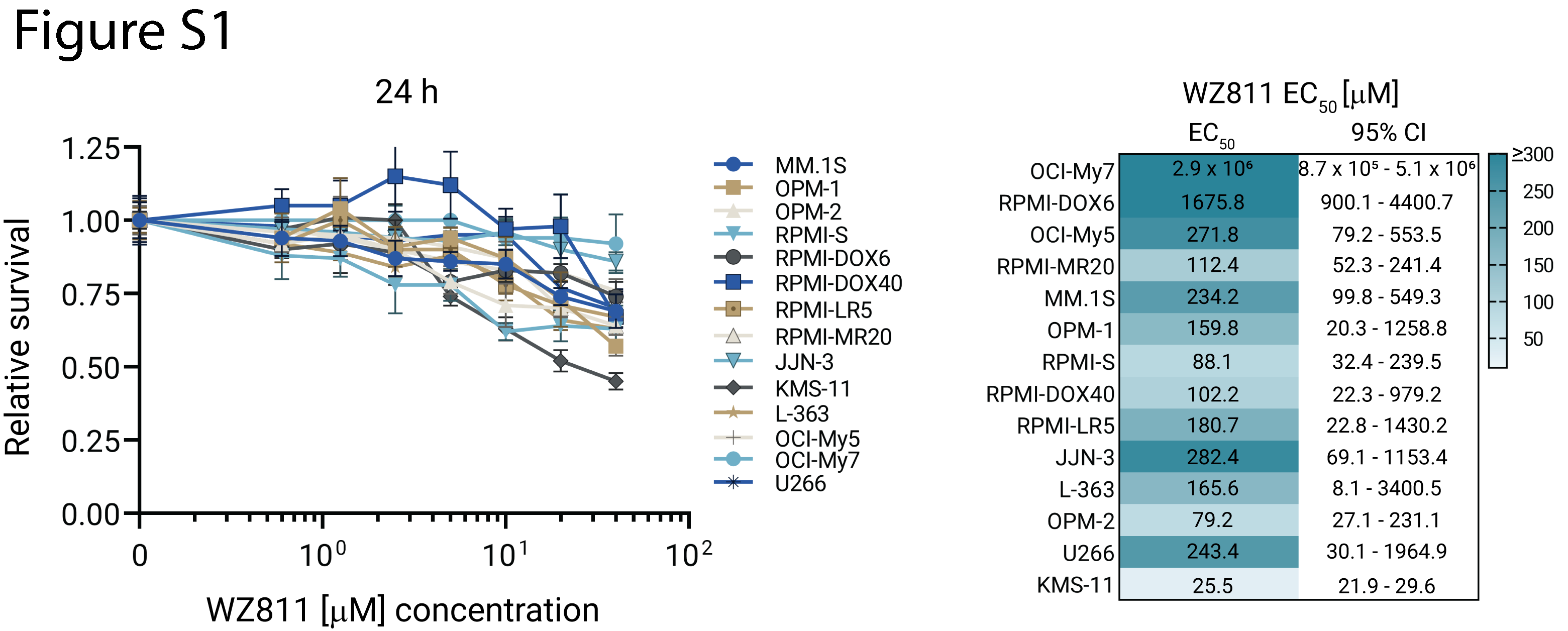

Supplement: Supplementary file 2 — High Resolution Image (TIF 1.60 MB) [file 109_2026_2650_MOESM1_ESM.tif]

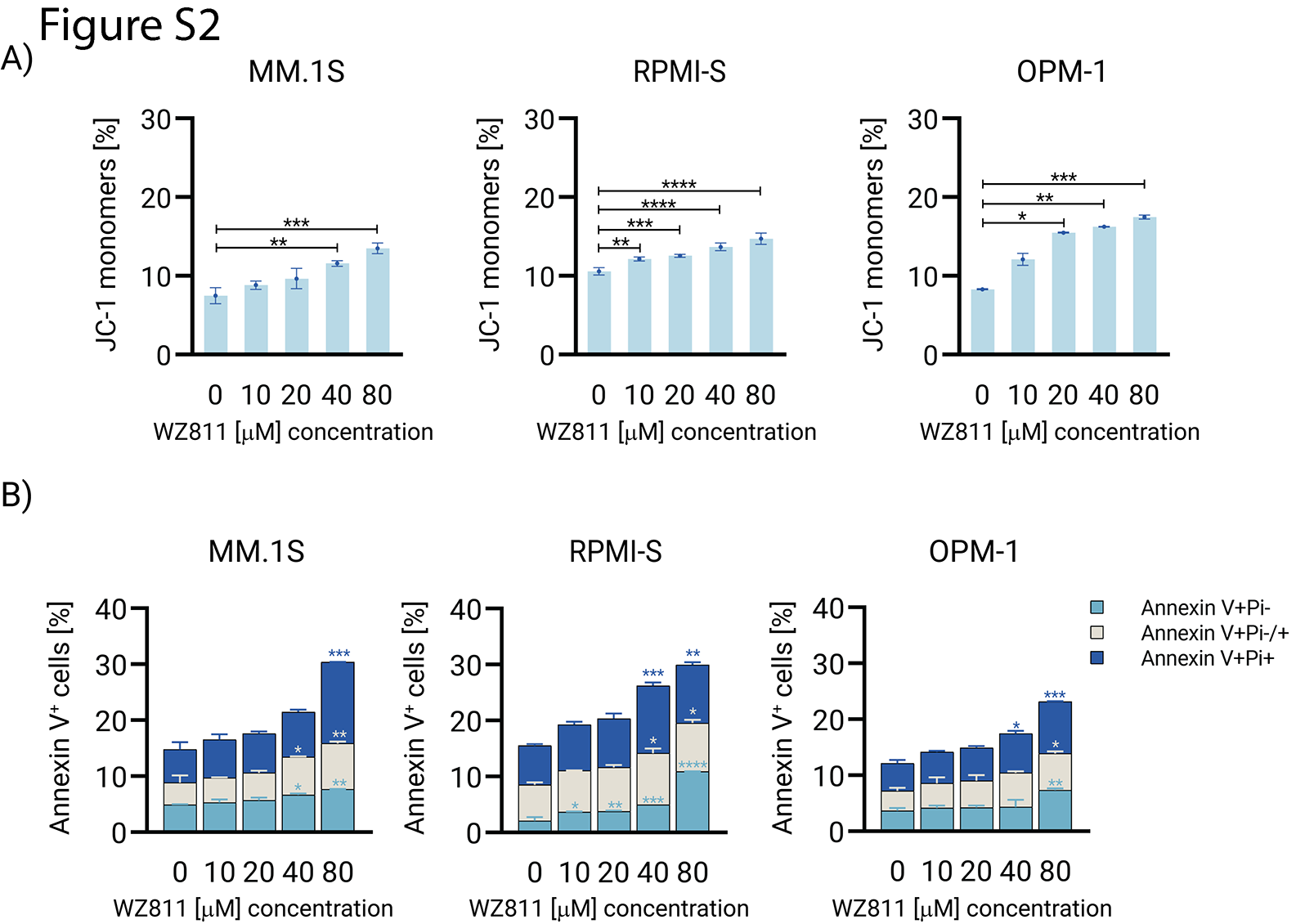

Supplement: Supplementary file 3 — (PNG 198 KB) [file 109_2026_2650_Fig9_ESM.png]

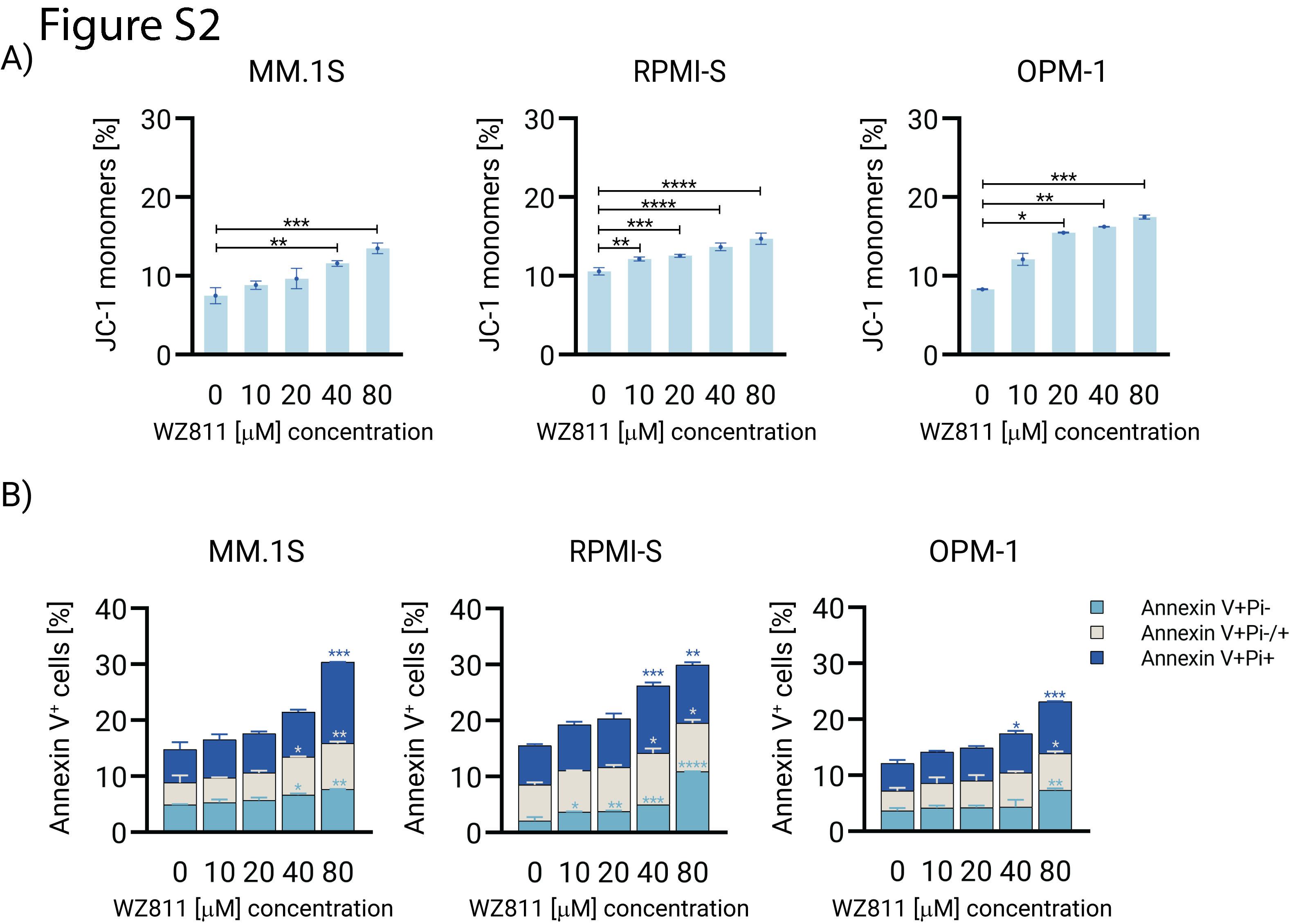

Supplement: Supplementary file 4 — High Resolution Image (TIF 1.96 MB) [file 109_2026_2650_MOESM2_ESM.tif]

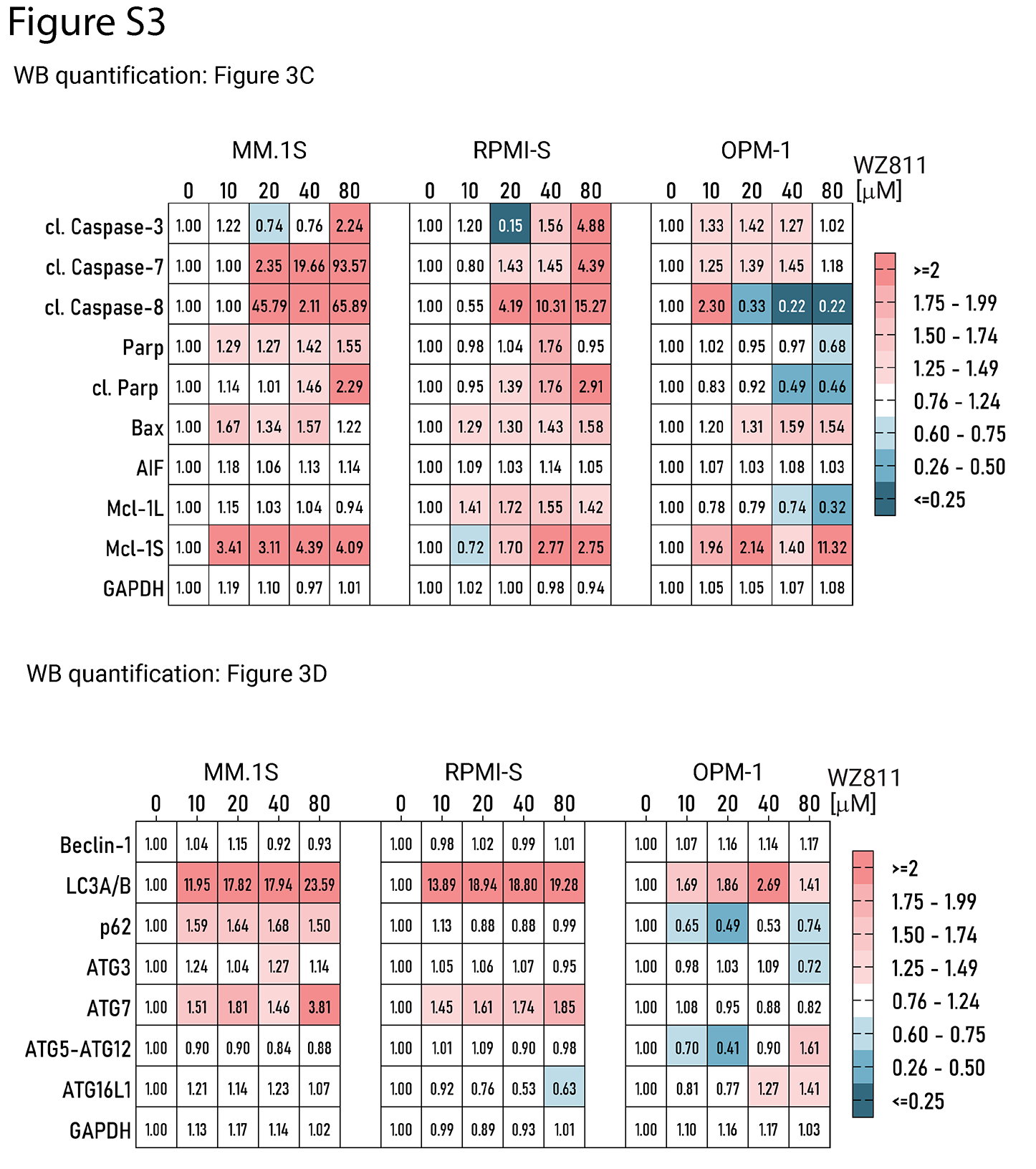

Supplement: Supplementary file 5 — (PNG 235 KB) [file 109_2026_2650_Fig10_ESM.png]

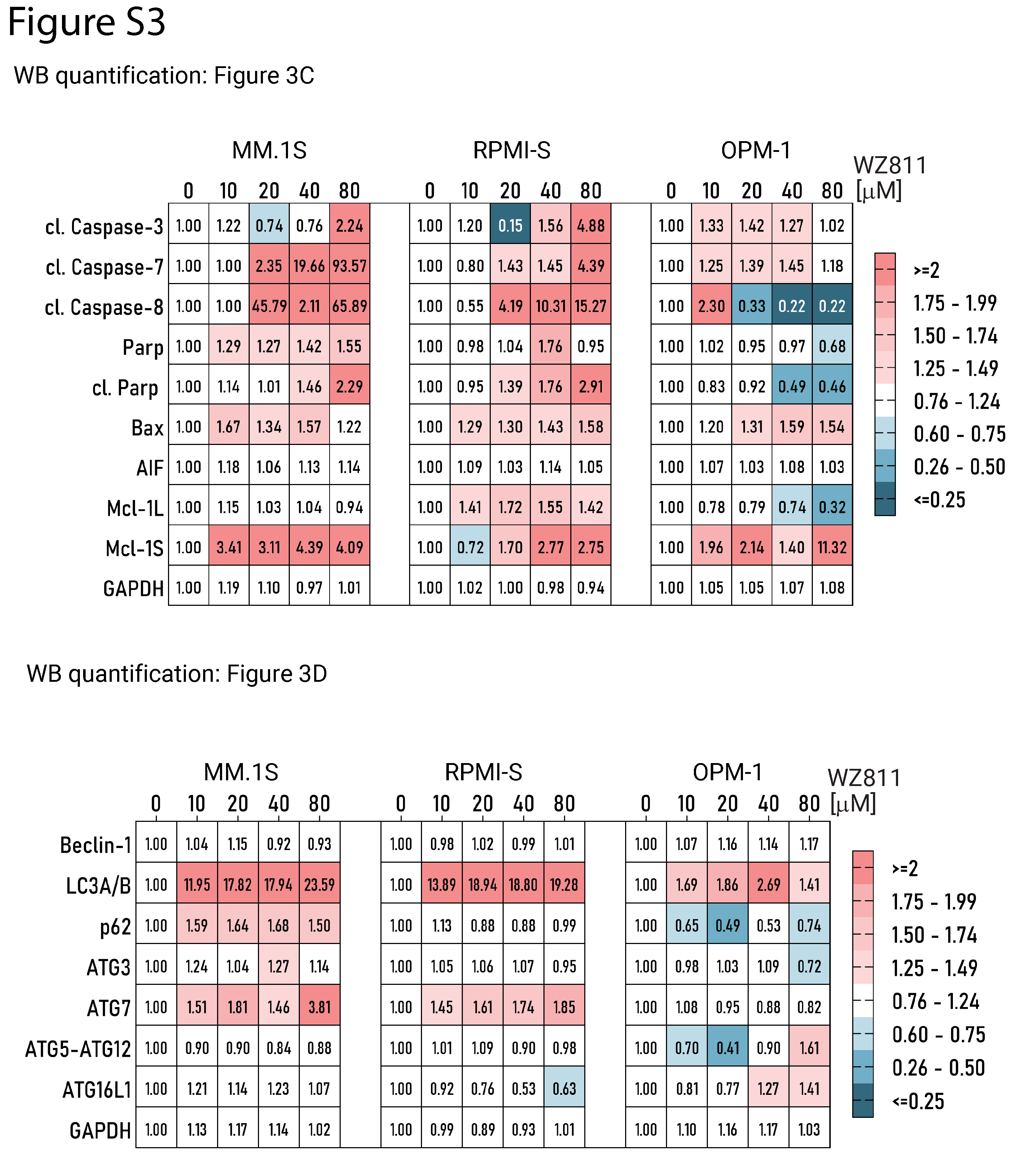

Supplement: Supplementary file 6 — High Resolution Image (TIF 3.05 MB) [file 109_2026_2650_MOESM3_ESM.tif]

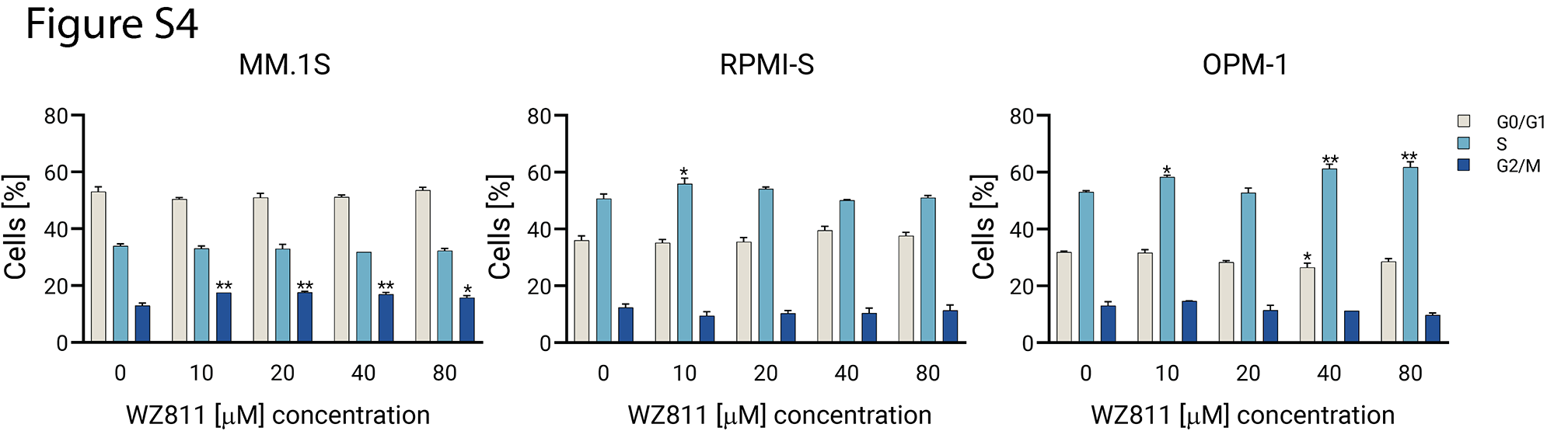

Supplement: Supplementary file 7 — (PNG 198 KB) [file 109_2026_2650_Fig11_ESM.png]

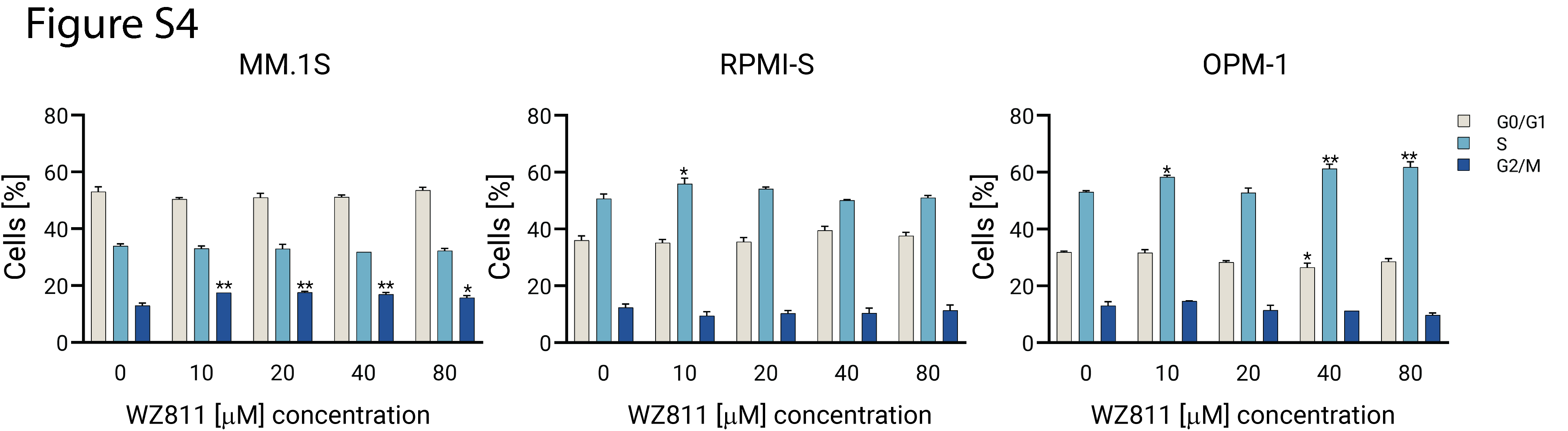

Supplement: Supplementary file 8 — High Resolution Image (TIF 1.15 MB) [file 109_2026_2650_MOESM4_ESM.tif]

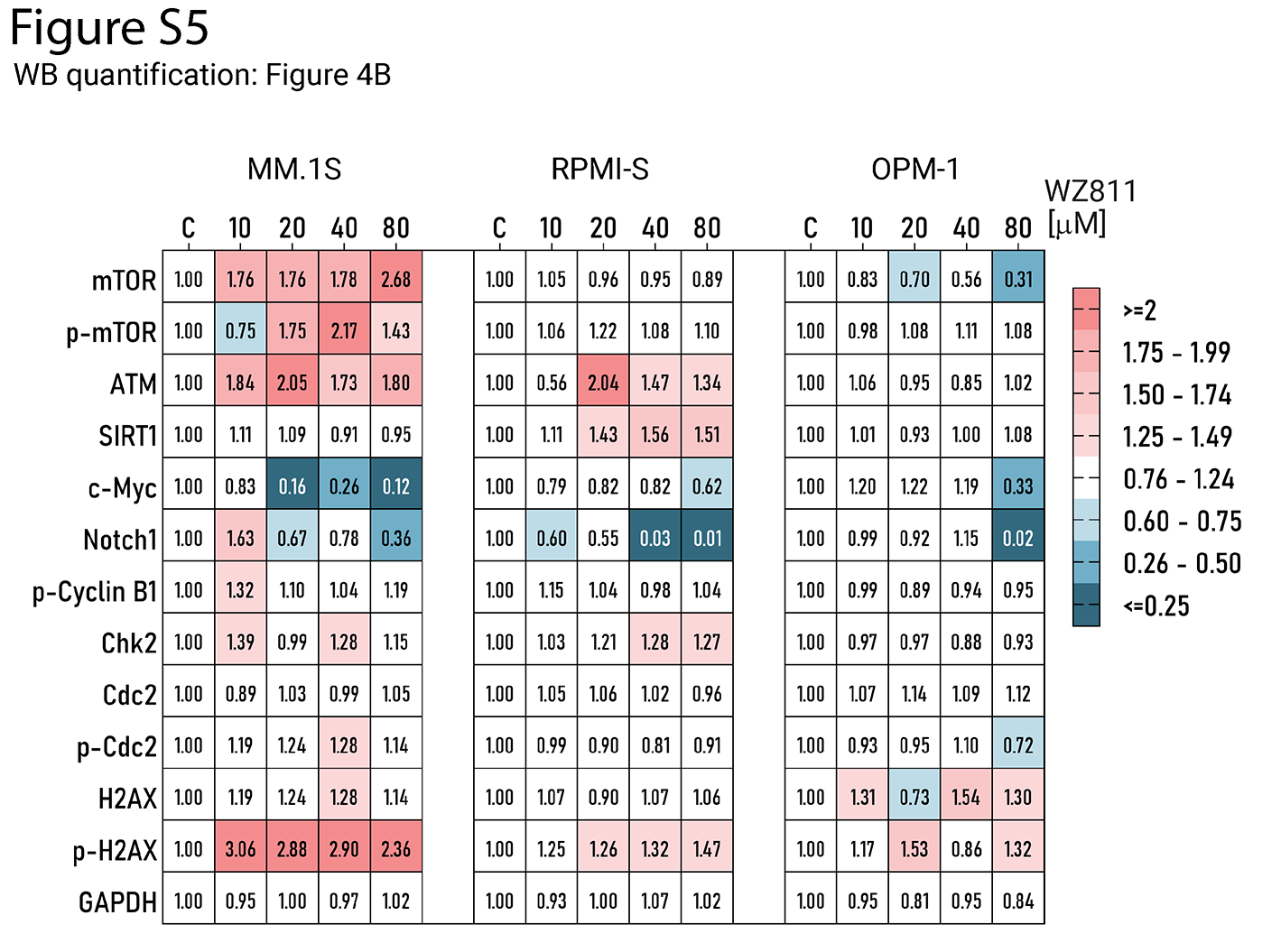

Supplement: Supplementary file 9 — (PNG 198 KB) [file 109_2026_2650_Fig12_ESM.png]

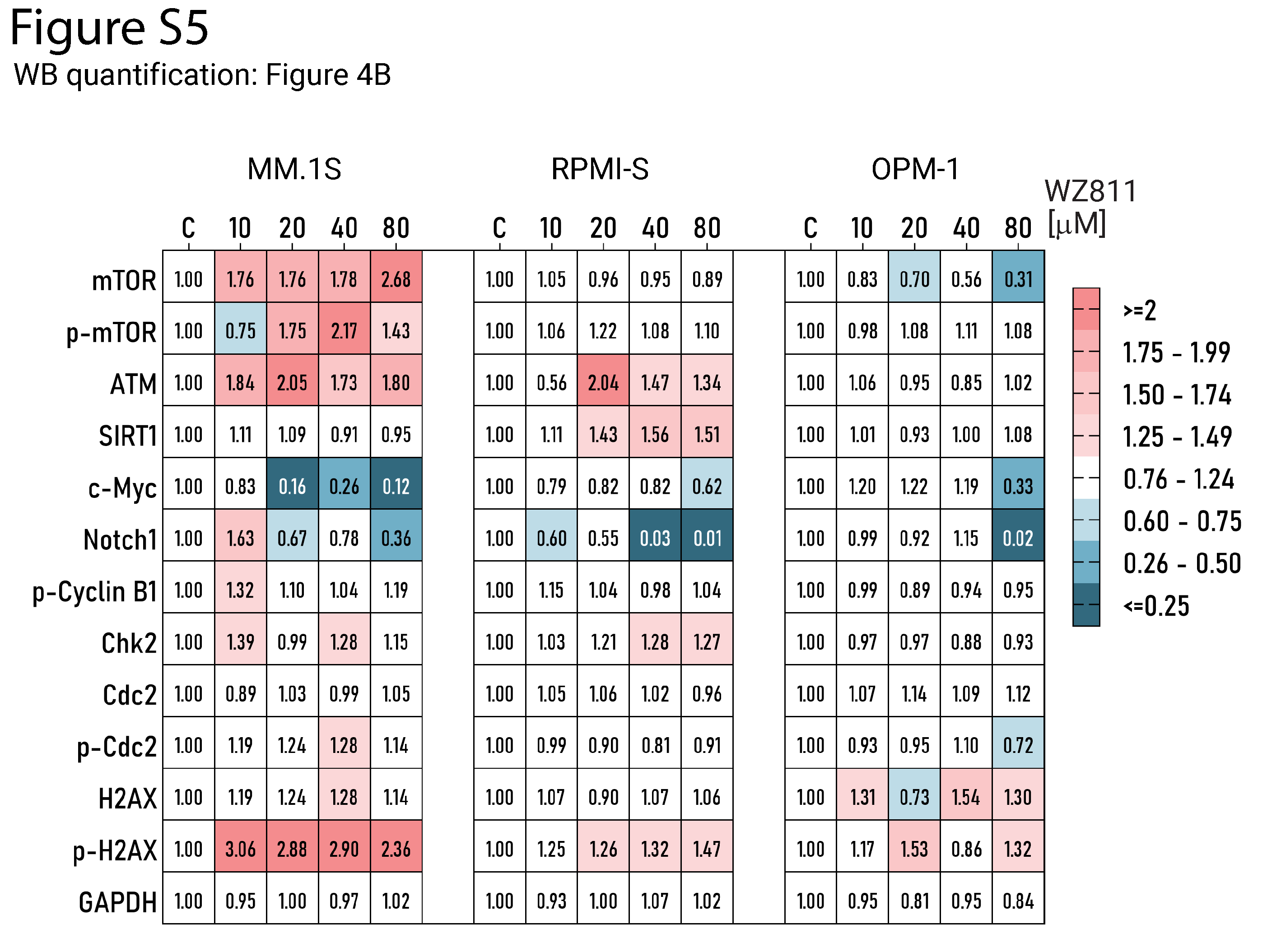

Supplement: Supplementary file 10 — High Resolution Image (TIF 2.07 MB) [file 109_2026_2650_MOESM5_ESM.tif]
