# Supplementary material for: Anti-myeloma activity of the CXCR4 antagonist WZ811
Source: J Mol Med (Berl). 2026 Feb 17;104(1):45. doi: 10.1007/s00109-026-02650-4 (PMC12913330; doi:10.1007/s00109-026-02650-4)
Supplement: Supplementary file 11 — (DOCX 19.1 KB) [file 109_2026_2650_MOESM6_ESM.docx]

**SUPPLEMENTARY FIGURE LEGENDS**

Supplementary Table 1: **Clinical and laboratory characteristics of primary MM samples treated *ex vivo* with WZ817.** Clinical data were collected from patients with a confirmed diagnosis of multiple myeloma and comprise: demographic information (age, sex), baseline biochemical parameters (serum M-protein, serum and urine free light chains, creatinine, calcium, hemoglobin, albumin, lactate dehydrogenase, platelet counts), prior treatment history (number of prior lines, and types of previous therapies), and cytogenetic/molecular abnormalities.

Supplementary Figure S1: **WZ811 decreases survival of MM cell lines.** MM cell lines (MM.1S, OPM-1, OPM-2, RPMI-S, RPMI-DOX6, RPMI-DOX40, RPMI-LR5, RPMI-MR20, JJN-3, KMS-11, L-363, OCI-My5, OCI-My7, and U266 cells) were treated with WZ811 at concentrations of 0.625, 1.25, 2.5, 5, 10, 20, and 40 μM for 24 h, and cell survival was assessed by MTT assay. The EC_50_ values of WZ811 were determined in MM cell lines for 24 h by the CalcuSyn software. Each treatment with a specific concentration of WZ811 was performed in triplicate. The presented data are mean ± standard deviation, expressed as survival/viability relative to untreated controls. EC_50_ (half maximal effective concentration) is shown with lower and upper 95% confidence intervals (CI).

Supplementary Figure S2: **WZ811 induces apoptotic cell death in MM cells.** MM.1S, RPMI-S, and OPM-1 cells were treated with indicated concentrations (10, 20, 40, and 80 μM) of WZ811 for 24 h. (A) Decrease of mitochondrial membrane potential in MM cells after exposure to WZ811 was determined by staining with the fluorescent JC1 dye, which resulted in increased levels of JC-1 monomers, and analyzed by a FACS Canto II flow cytometer. (B) Induction of early apoptotic (Annexin V+/Pi−), late apoptotic (Annexin V+/Pi+/−), and necrotic (Annexin V+/Pi+) cells was evaluated with Annexin V-FITC and PI staining and analyzed by a FACS Canto II flow cytometer. The data are from three independent experiments and presented as means ± standard deviation. Significant differences between treatments and control were identified by One-way ANOVA followed by Dunnett's multiple comparison test with *p < 0.05, **p < 0.01, ***p < 0.001, and ****p < 0.0001.

Supplementary Figure S3: **Quantification of western blot analysis.** Densitometric quantification of the western blot analysis shown in Figures 3C and 3D was performed using ImageJ software to evaluate protein expression levels.

Supplementary Figure S4: **WZ811 induces cell cycle arrest in MM cells.** MM.1S, RPMI-S, and OPM-1 cells were exposed to indicated concentrations (10, 20, 40, and 80 μM) of WZ811 for 24 h, and their cell cycle profiles were analyzed using propidium iodide (Pi) staining. The distribution of cells in G_0_/G_1_, S, and G_2_/M phase was measured by a FACS Canto II flow cytometer and analyzed with *De Novo* FCS Express software. The data are from three independent experiments and are presented as means ± standard deviation. Significant differences between treatments and control were identified by One-way ANOVA followed by Dunnett's multiple comparison test with *p < 0.05 and **p < 0.01.

Supplementary Figure S5: **Quantification of western blot analysis.** Densitometric quantification of the western blot analysis shown in Figure 4B was performed using ImageJ software to evaluate protein expression levels.
