# Supplementary material for: Anti-myeloma activity of the CXCR4 antagonist WZ811
Source: J Mol Med (Berl). 2026 Feb 17;104(1):45. doi: 10.1007/s00109-026-02650-4 (PMC12913330; doi:10.1007/s00109-026-02650-4)
Supplement: Supplementary file 12 — (DOCX 25.4 KB) [file 109_2026_2650_MOESM7_ESM.docx]

**SUPPLEMENTARY METHODS**

**Multiple myeloma (MM) cell lines**

In our study, we used a panel of 14 MM cell lines: MM.1S, OPM-1, OPM-2, RPMI-S, RPMI-DOX6, RPMI-DOX40, RPMI-LR5, RPMI-MR20, JJN-3, KMS-11, L-363, OCI-My5, OCI-My7, and U266 cells). This panel spans MM disease stages from steroid-sensitive (e.g., MM.1S, derived from pleural effusion of advanced MM patient, sensitive to glucocorticoids) to relapsed/refractory models (e.g., RPMI-DOX6/40, DOX-resistant; RPMI-LR5/MR20, melphalan and also lenalidomide/mitoxantrone-resistant variants of RPMI-S). MM.1S cells are hypodiploid and IL-6 sensitive with low-to-moderate aggressiveness. OPM-1 cells show complex cytogenetics with 1q gain and moderate aggressiveness, while OPM-2 cells harbor t(4;14) and display a highly proliferative phenotype. RPMI-S (8226) cells are hypotetraploid with del(13q) and represent a moderate baseline model, whereas drug-resistant variants RPMI-DOX6 and RPMI-DOX40 (del17p/TP53 loss), and RPMI-LR5 and RPMI-MR20 exhibit high-risk, refractory phenotypes. Additional high-risk models included JJN-3 and KMS-11 cells with t(4;14)-associated abnormalities. L-363 cells are near-diploid with moderate aggressiveness, OCI-My5 and OCI-My7 cells exhibit complex, highly proliferative profiles, and U266 cells carry t(11;14) with NRAS mutation and show moderate-to-high aggressiveness. All MM cell lines were cultured in RPMI 1640 medium (Cellgro, Mediatech, VA) supplemented with 10% heat-inactivated fetal bovine serum (FBS; Harlan, Indianapolis, IN), 100 U/ml penicillin, 100 μg/ml streptomycin, and 2 mM L-glutamine (GIBCO, Grand Island, NY) at 37°C in a 5% CO2 atmosphere.

The HS-5 stromal cell line was cultured in Dulbecco’s modified Eagle medium (DMEM; Cellgro, Mediatech, VA) supplemented with 10% heat-inactivated fetal bovine serum (FBS; Harlan, Indianapolis, IN), 100 u/ml penicillin, 100 μg/ml streptomycin and 2 mM L-glutamine (GIBCO, Grand Island, NY) at 37°C in 5% CO2. Cell lines were authenticated by cell profiling (Promega PowerPlex Fusion System kit; Promega, Madison, WI) in the past three years. The presence of mycoplasma was periodically tested by PCR.

**Drug viability assays**

The inhibitory effects of WZ811, both alone and in combination with conventional or novel anti-multiple myeloma (MM) agents, on the survival of MM cell lines were assessed using the 3-[4,5-dimethylthiazol-2-yl]-2,5-diphenyltetrazolium bromide (MTT) assay (Sigma-Aldrich, St. Louis, MO). MM cell lines were plated in 96-well plates at a density of 10,000 cells per well and treated with increasing concentrations of WZ811 for 24, 48, and 72 hours. After treatment, formazan crystals were dissolved by adding 150 μl of dimethyl sulfoxide (DMSO), and absorbance was measured at 540 nm and 690 nm using a microplate reader (Dynatech Lab Inc., Chantilly, VA).

Purified MM patient cells and peripheral blood mononuclear cells (PBMCs) were seeded in 384-well plates at a density of 10,000 cells per well and treated with increasing concentrations of WZ811 for 48 hours at 37°C. Cell viability was determined using the CellTiter-Glo (CTG) assay (Promega, Madison, WI). After adding CTG, the plates were incubated for 30 minutes and read using a Luminoskan luminometer (Labsystems, Franklin, MA).

The concentration of the drug that inhibited cell survival by 50% (EC50) was calculated using Calcusyn software (Biosoft, Ferguson, MO). To evaluate the interaction between WZ811 and other anti-MM agents, isobologram analysis and calculation of the combination index (CI) were performed using the Calcusyn software according to the Chou-Talalay method. A CI value of 1 indicated an additive effect, a CI value less than 1 indicated synergism, and a CI value greater than 1 indicated antagonism.

**Flow cytometric analysis of apoptosis and cell cycle**

***Detection of apoptosis***

Apoptotic cells were quantified using the Annexin V-FITC/PI apoptosis assay. Briefly, both suspension and adherent cells were collected and washed twice with cold PBS. Subsequently, 3 × 10^5 cells were resuspended in 100 μl of the manufacturer-supplied 1X binding buffer and mixed with 5 μl of Annexin V-FITC (BD Biosciences Pharmingen, San Diego, CA) and 5 μl of propidium iodide (PI). After a 30-minute incubation in the dark at room temperature (RT), cells were analyzed by a FACS Canto II flow cytometer (Becton Dickinson, Mountain View, CA) using a 96-well format.

***Cytofluorimetric analysis of mitochondrial membrane potential***

The mitochondrial membrane potential of WZ811-treated cells, as well as control MM cells, was assessed using the JC-1 fluorescent probe. JC-1 is a mitochondria-selective dye that forms aggregates in normal, polarized mitochondria, emitting an orange fluorescence. In cells with depolarized mitochondrial membranes, JC-1 remains in its monomeric form, emitting green fluorescence. Briefly, 3 × 10^5 cells were incubated in 200 μl of PBS/0.2% BSA containing 4 μM JC-1 (Molecular Probes, Eugene, OR) for 30 minutes at 37°C in the dark. Following the incubation, cells were analyzed using a FACS Canto II flow cytometer (Becton Dickinson, Mountain View, CA) in a 96-well format.

***Cell cycle analysis***

Changes in the cell cycle status of WZ811-treated cells were assessed by flow cytometric measurement of DNA content using PI staining. Briefly, MM cells (3 × 10^5) were collected, washed twice with cold PBS, and incubated with 0.05% Triton X-100 and 15 μl of RNAse A (10 mg/ml) for 20 minutes at 37°C. Following incubation, the cells were cooled on ice for at least 10 minutes before the addition of PI (50 μg/ml). The stained cells were then analyzed using a FACS Canto II flow cytometer (Becton Dickinson, Mountain View, CA) in a 96-well format.

***Flow cytometry measurements and data analysis***

Flow cytometry was performed using a FACS Canto II flow cytometer equipped with a 488 nm excitation laser. Fluorochromes were excited with the 488 nm laser and data were collected through the following photomultipliers: Annexin V-FITC and PI (FL1, FL3); JC-1 (FL1, FL2, with the ratio FL2/FL1); and cell cycle (log FL3 for sub-G1, linear FL2 for the DNA cell cycle histogram, and FL3 peak *versus* integral for doublet discrimination). Forward and side scatter characteristics were used to exclude cell debris from the analysis. For each analysis, 10,000 to 20,000 cells were acquired. Data were analyzed using De Novo FCS Express software (De Novo Software, Los Angeles, CA).

**Co-culture model using CFSE assay**

To distinguish MM cells in co-culture models, MM cells were labeled with carboxyfluorescein diacetate succinimidyl ester (CFSE), a fluorescent dye that evenly distributes between daughter cells during cell division. The labeled MM cells were then seeded onto unlabeled bone marrow stromal HS-5 cells and cultured for 24 and 72 hours. Changes in the mean fluorescence intensity of the CFSE signal served as a surrogate marker for cell proliferation. Briefly, MM cells were labeled with 1 μM CFSE (Molecular Probes, Eugene, OR) for 10 minutes at 37°C in serum-free RPMI in the dark. The labeling reaction was terminated by adding RPMI 1640 medium supplemented with 2% FBS, and the cells were washed three times with RPMI 1640 containing 10% FBS. Subsequently, the cells were seeded either alone or together with unlabeled bone marrow stromal HS-5 cells, which had been seeded 24 hours prior to co-culture. Drug-based studies were then conducted on these co-culture systems.

**Flow cytometry immunofluorescence analysis**

Both MM cells, labeled with CFSE alone or in coculture with unlabeled bone marrow stromal cells (HS-5), were collected and washed with PBS containing 0.5% BSA. Before staining with surface marker antibodies, cells were incubated with 5 μl of Fc receptor blocking solution (BioLegend, San Diego, CA) for 10 minutes at RT to block non-specific binding. After blocking, cells were incubated with primary antibodies directed at CXCL12, laminin, and collagen IV (Sigma-Aldrich, St. Louis, MO, USA) in a final volume of 100 μl for 30 minutes at RT, followed by incubation with allophycocyanin (APC) fluorescence-tagged secondary antibody (goat anti-mouse IgG; Molecular Probes, Eugene, OR) for 1 hour at RT in the dark. After several washes with PBS, 7-aminoactinomycin D (7-AAD; Molecular Probes, Eugene, OR) was added to the cells at a final concentration of 1 μg/mL in PBS to gate only viable cells. Control cells were stained with secondary antibody alone to detect non-specific interactions. After staining, cells were washed in PBS and then analyzed on a FACS Aria Special Sorter (Becton Dickinson, Mountain View, CA).

**Side population analysis by Hoechst 33342 assay**

For side population analysis, cells were labeled with Hoechst 33342 dye following the method described by Goodell et al., with some modifications. Initially, cells were washed in pre-warmed RPMI 1640 containing 2% FBS and 10 mmol/L Hepes buffer (Life Technologies, Carlsbad, CA, USA). Subsequently, they were resuspended in RPMI 1640 supplemented with 2% FBS and 10 mmol/L Hepes buffer, along with 5 μg/mL of Hoechst 33342 dye (Molecular Probes, Eugene, OR), and incubated for 90 minutes at 37°C with intermittent shaking. To ensure specificity, a negative control was prepared by preincubating cells with 50 μmol/L of reserpine, an ABC transporter inhibitor. After the incubation period, cells were washed with ice-cold PBS containing 2% FBS and 10 mmol/L Hepes buffer. To gate only viable cells, 7-aminoactinomycin D (7-AAD; Molecular Probes, Eugene, OR) was added to the cells at a final concentration of 1 μg/mL in PBS with 2% FBS and 10 mmol/L Hepes buffer. The labeled cells were then analyzed using a FACS Aria Special Sorter equipped with a UV laser (Becton Dickinson, Mountain View, CA). Hoechst 33342 dye was excited at 357 nm, followed by dual-wavelength fluorescence analysis (blue, 402 - 446 nm; red, 650 - 670 nm).

**Western immunoblotting analysis**

Following treatment with WZ811, cells were washed twice with ice-cold PBS and resuspended in 100 μl of ice-cold cell lysis buffer (1% Nonidet P-40, 50 mM Tris, pH 7.4, 150 mM NaCl, 2 mM EDTA, 2 mM PMSF, 1 mM sodium vanadate, 1 mM sodium fluoride, and a protease inhibitor mixture). The cells were then incubated on ice for 20 minutes. After centrifugation at 10,000 × g for 10 minutes, the supernatants were collected, and protein concentrations were measured using a Bradford protein assay kit. Equal amounts of protein (20 μg) were mixed with 4X SDS-PAGE sample buffer (Invitrogen, Carlsbad, CA) and 10X reducing agent (0.5 M dithiothreitol, Invitrogen, Carlsbad, CA), then separated by SDS-PAGE. The resolved proteins were transferred to a nitrocellulose membrane (Bio-Rad) using a semi-dry transfer system. Membranes were blocked for 1 hour at room temperature with 5% non-fat dry milk in Tris-buffered saline (TBS), pH 7.4, containing 1% Tween 20 (TBS-T). After blocking, the membranes were incubated overnight at 4°C with primary antibodies diluted 1:1000. The primary antibodies used included anti-caspase-3, -caspase-7, -caspase-8, -PARP, -Bax, -AIF, -Mcl-1, -Beclin-1, -LC3A/B, -SQSTM1/p62, -ATG3, -ATG7, -ATG5-12, -ATG16L1, -mTOR, -p-mTOR, -ATM, -SIRT1, -c-Myc, -Notch1, -p-Cyclin B1, -Chk2, -Cdc2, -p-Cdc2, -histone H2AX (H2AX), -p-histone H2AX (p-H2AX), and -GAPDH (Cell Signaling Technology, Danvers, MA). Membranes were then washed with TBS-T and incubated with horseradish peroxidase-conjugated goat anti-mouse or anti-rabbit secondary antibodies (Cell Signaling Technology, Danvers, MA) for 1 hour at room temperature. Proteins were visualized using the enhanced chemiluminescent (ECL) system (Amersham Bioscience, Little Chalfont, UK).

**RNA extraction, cDNA synthesis, and quantitative real-time PCR (qRT-PCR) analysis**

Total RNA from MM cell lines treated with WZ811 was extracted using TRIzol reagent (Ambion, Austin, TX) according to the manufacturer’s protocol. Glycogen was added as a carrier at a final concentration of 5 µg/µl for each reaction. The integrity of the isolated RNA samples was assessed using the QIAxpert system (Qiagen, Hilden, Germany). Isolated RNA was then used for cDNA synthesis with the High Capacity RNA-to-cDNA kit (Applied Biosystems, Waltham, MA). The cDNA synthesis reaction (20 μl total volume) included the following components: 10 μl of 2x RT Buffer, 1 μl of 20x RT Enzyme Mix, and 9 μl of the RNA sample. Reverse transcription was performed using the C1000 Touch thermal cycler (Bio-RAD, Hercules, CA) under the following conditions: 37°C for 60 minutes, followed by 95°C for 5 minutes. The resulting cDNA was used as a template for quantitative real-time PCR (qRT-PCR) using specific TaqMan Gene Expression Assays to detect CXCR4 (Assay ID: Hs00607978_s1) and GAPDH (Assay ID: Hs02758991_g1). The components of the qPCR reaction (total volume 10 μl) were as follows: 5 μl of 2x TaqMan Gene Expression Master Mix, 0.5 μl of 20x TaqMan Gene Expression Assay, 3.5 μl of RNase-free water, and 1 μl of cDNA template. The qRT-PCR was performed in triplicate on an AriaMx Real-Time PCR instrument (Agilent Technologies, Santa Clara, CA). The PCR cycling conditions included an initial UDG incubation at 50°C for 2 minutes, enzyme activation at 95°C for 10 minutes, followed by 40 cycles of denaturation at 95°C for 15 seconds and amplification at 60°C for 1 minute. The relative mRNA expression was calculated using the 2^(-ΔΔCt) comparative CT method, normalized to GAPDH and the control.

**Tumor cell implantation in mouse model**

Mice were housed in the Animal Research Facility of the Biomedical Research Center SAS, and all experiments were conducted in accordance with approved protocols. Both male and female mice aged five to six weeks were used in this study, and all animals were obtained from Charles River Laboratories. In the xenograft murine myeloma model, sublethally irradiated (150 rads) CB17/SCID mice were injected subcutaneously with MM.1S cells (2.5 × 10^6 cells per mouse) resuspended in PBS, 24 hours post-irradiation. Following engraftment, tumor-bearing mice were randomly assigned to two groups (six mice per group). The control group received the vehicle only (water with 10% DMSO and 5% Tween 80), while the treatment group received WZ811 at a concentration of 40 mg/kg (diluted in water for injection with 10% DMSO and 5% Tween 80). Treatments were administered by oral gavage once a day for 5 consecutive days, followed by 2 days off, and this cycle was repeated for 4 weeks. Mice were monitored every 2-3 days for changes in tumor burden, measured using calipers, and for body weight. Mice were sacrificed following institutional guidelines when tumors reached 2 cm or if the mice appeared moribund, to prevent unnecessary morbidity. Tumor volumes were calculated using the formula for the volume of an ellipsoid: V = 4/3π × (a/2) × (b/2)2, where "a" and "b" correspond to the longest and shortest diameters of the tumor, respectively.
