# Supplementary material for: Anti-myeloma activity of the CXCR4 antagonist WZ811
Source: J Mol Med (Berl). 2026 Feb 17;104(1):45. doi: 10.1007/s00109-026-02650-4 (PMC12913330; doi:10.1007/s00109-026-02650-4)
Supplement: Supplementary file 13 — (PDF 14.5 MB) [file 109_2026_2650_MOESM8_ESM.pdf]

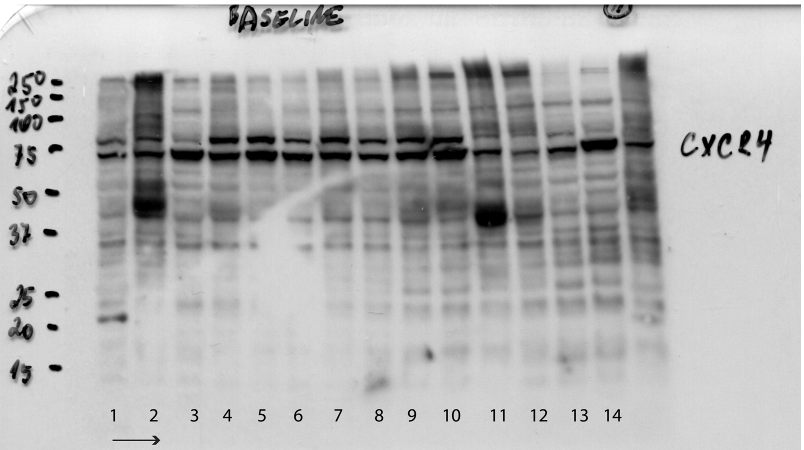

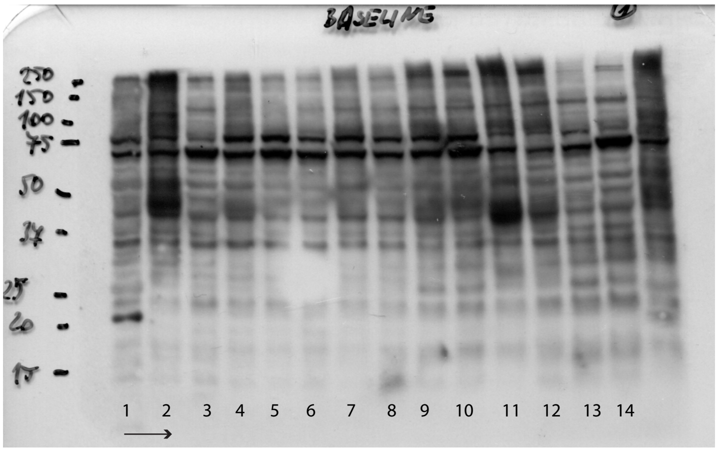

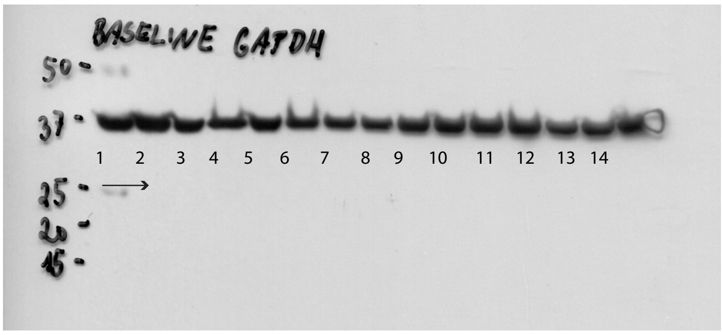

1 MM.1S
2 OPM-1
3 OPM-2
4 RPMI-S
5 RPMI-DOX6
6 RPMI-DOX40
7 RPMI-LR5
8 RPMI-MR20
9 JJN-3
10 KMS-11
11 L-363
12 OCI-My5
13 OCI-My7
14 U266

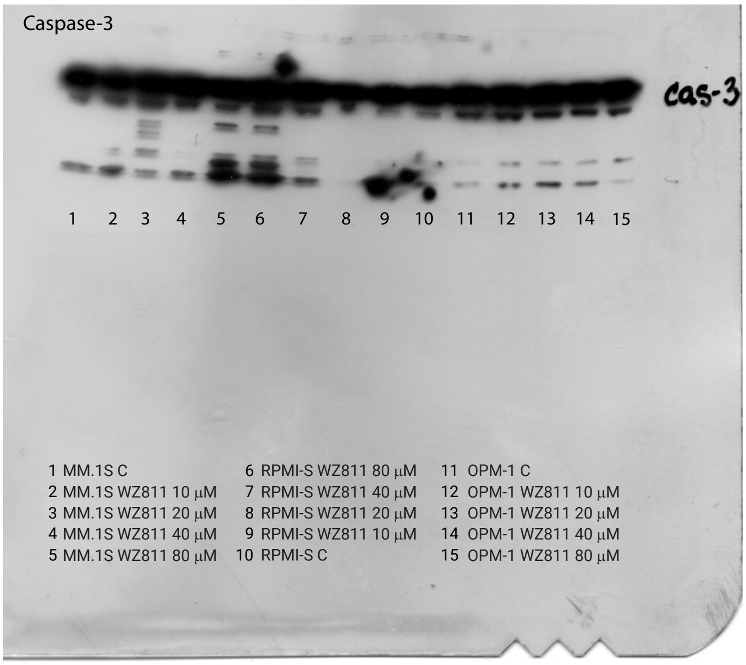

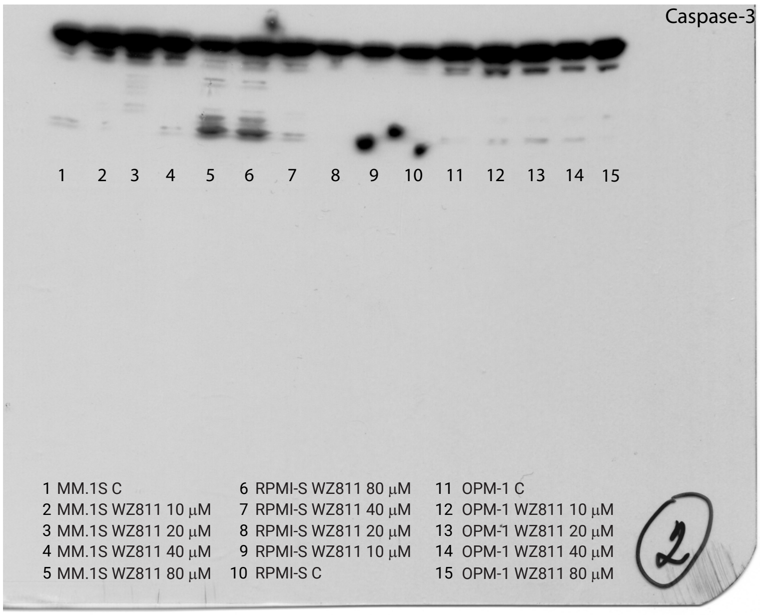

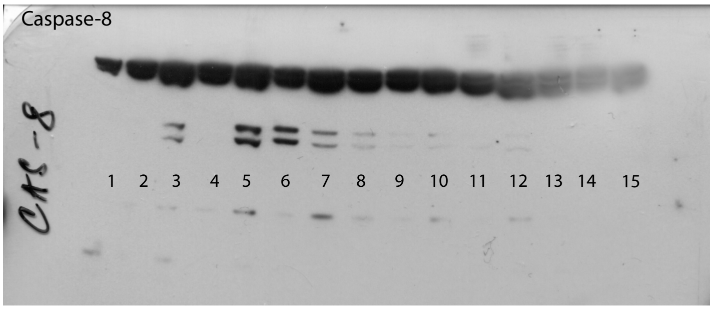

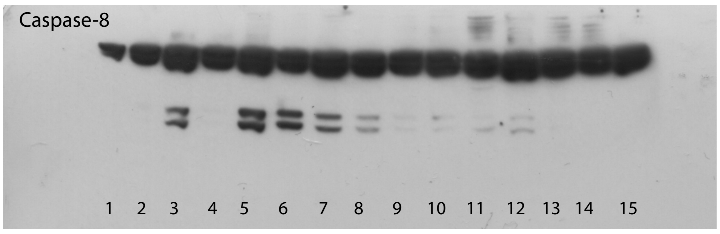

1 MM.1S C
2 MM.1S WZ811 10 µM
3 MM.1S WZ811 20 µM
4 MM.1S WZ811 40 µM
5 MM.1S WZ811 80 µM
6 RPMI-S WZ811 80 µM
7 RPMI-S WZ811 40 µM
8 RPMI-S WZ811 20 µM
9 RPMI-S WZ811 10 µM
10 RPMI-S C
11 OPM-1 C
12 OPM-1 WZ811 10 µM
13 OPM-1 WZ811 20 µM
14 OPM-1 WZ811 40 µM
15 OPM-1 WZ811 80 µM

1 MM.1S C
2 MM.1S WZ811 10 µM
3 MM.1S WZ811 20 µM
4 MM.1S WZ811 40 µM
5 MM.1S WZ811 80 µM
6 RPMI-S WZ811 80 µM
7 RPMI-S WZ811 40 µM
8 RPMI-S WZ811 20 µM
9 RPMI-S WZ811 10 µM
10 RPMI-S C
11 OPM-1 C
12 OPM-1 WZ811 10 µM
13 OPM-1 WZ811 20 µM
14 OPM-1 WZ811 40 µM
15 OPM-1 WZ811 80 µM

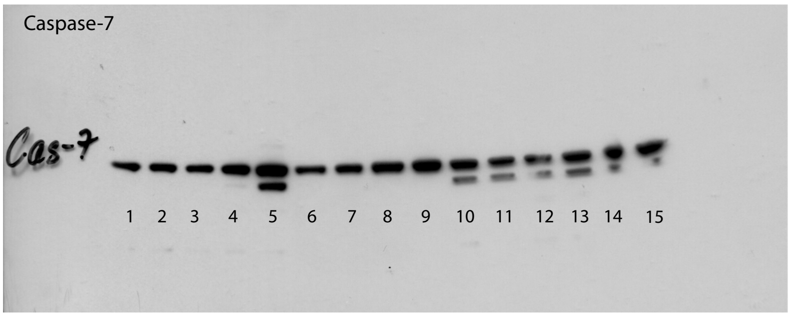

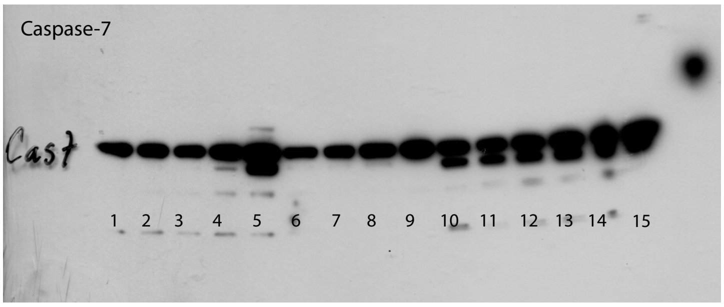

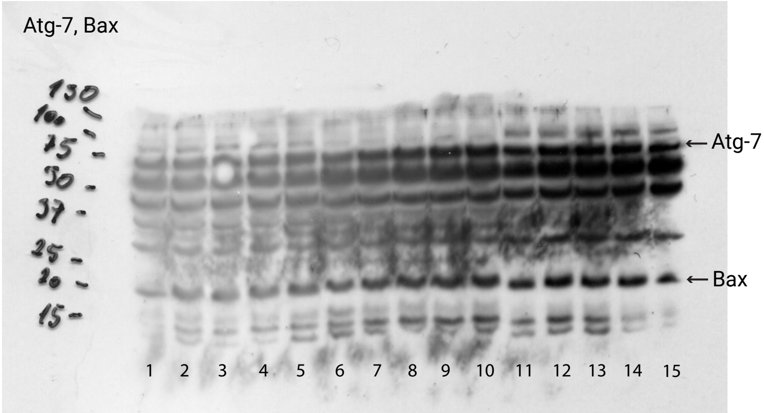

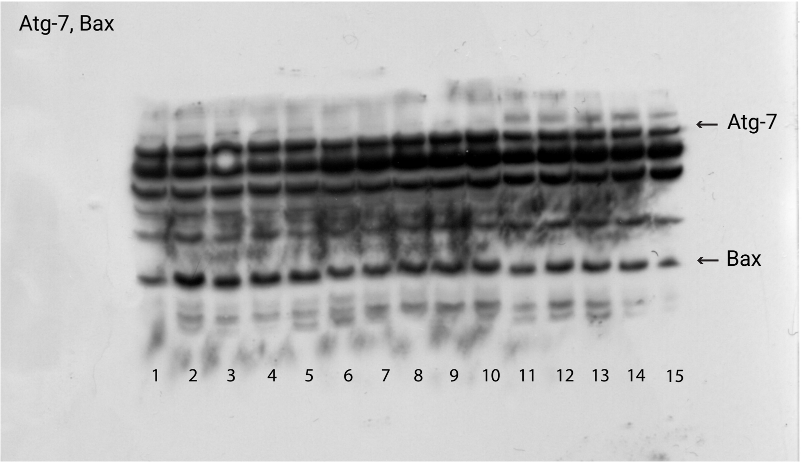

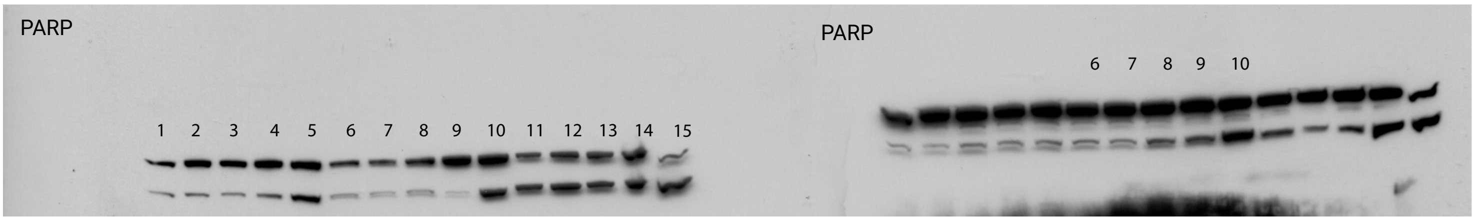

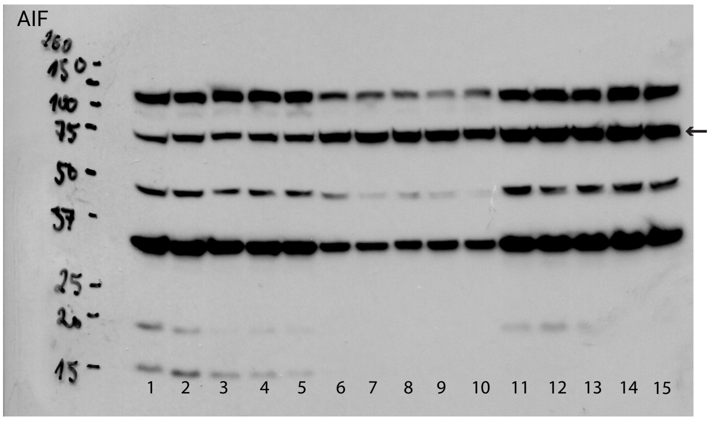

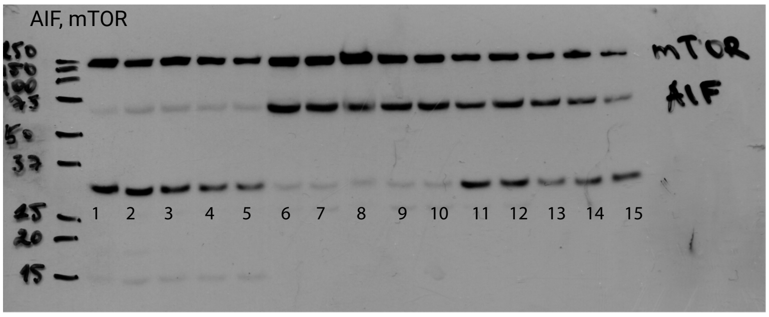

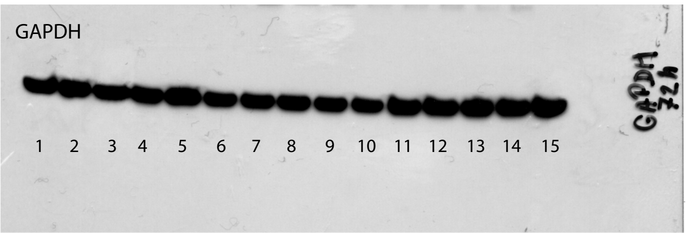

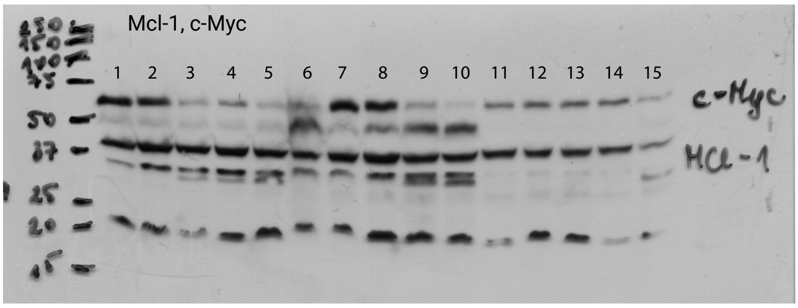

1 MM.1S C	6 RPMI-S C	11 OPM-1 C
2 MM.1S WZ811 10 μM	7 RPMI-S WZ811 10 μM	12 OPM-1 WZ811 10 μM
3 MM.1S WZ811 20 μM	8 RPMI-S WZ811 20 μM	13 OPM-1 WZ811 20 μM
4 MM.1S WZ811 40 μM	9 RPMI-S WZ811 40 μM	14 OPM-1 WZ811 40 μM
5 MM.1S WZ811 80 μM	10 RPMI-S WZ811 80 μM	15 OPM-1 WZ811 80 μM

1 MM.1S C	6 RPMI-S C	11 OPM-1 C
2 MM.1S WZ811 10 μM	7 RPMI-S WZ811 10 μM	12 OPM-1 WZ811 10 μM
3 MM.1S WZ811 20 μM	8 RPMI-S WZ811 20 μM	13 OPM-1 WZ811 20 μM
4 MM.1S WZ811 40 μM	9 RPMI-S WZ811 40 μM	14 OPM-1 WZ811 40 μM
5 MM.1S WZ811 80 μM	10 RPMI-S WZ811 80 μM	15 OPM-1 WZ811 80 μM

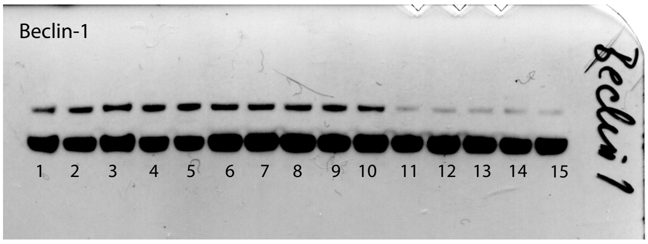

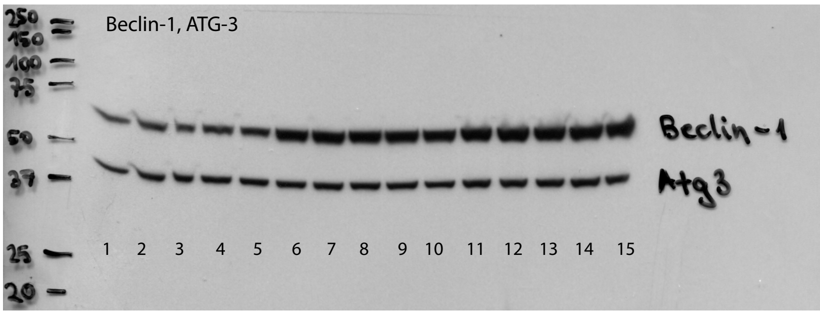

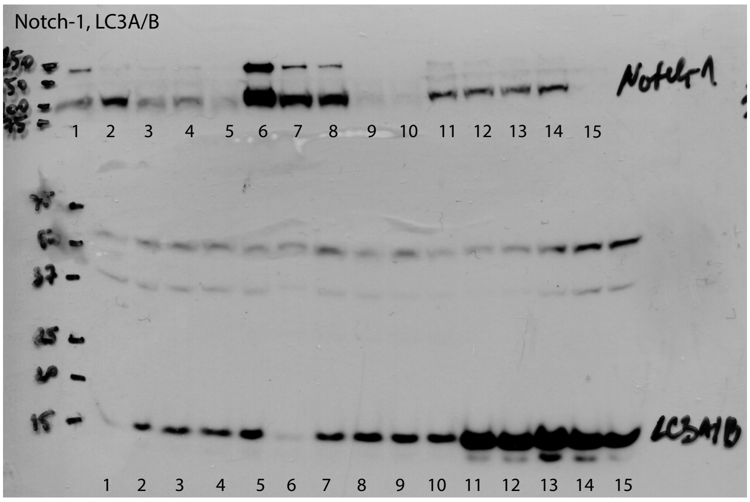

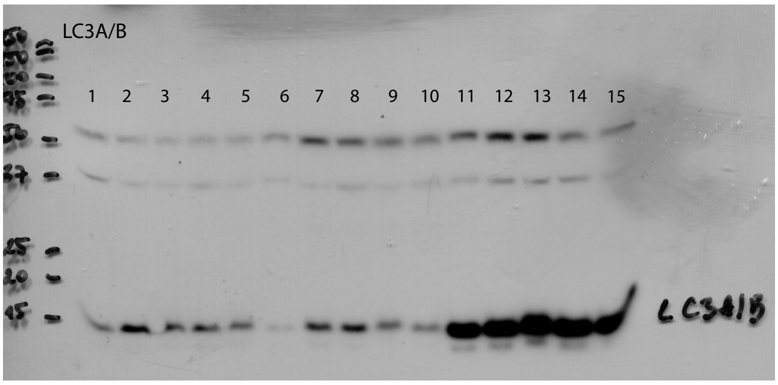

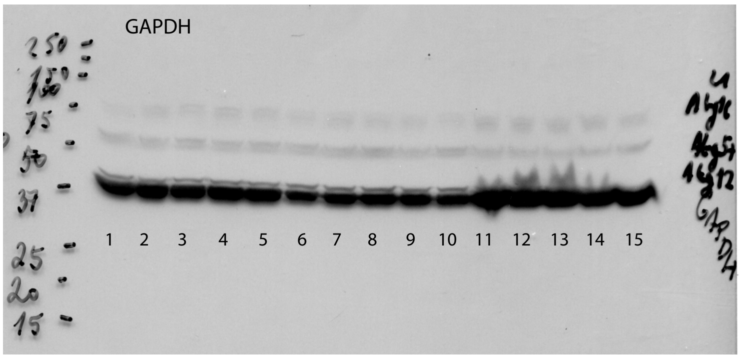

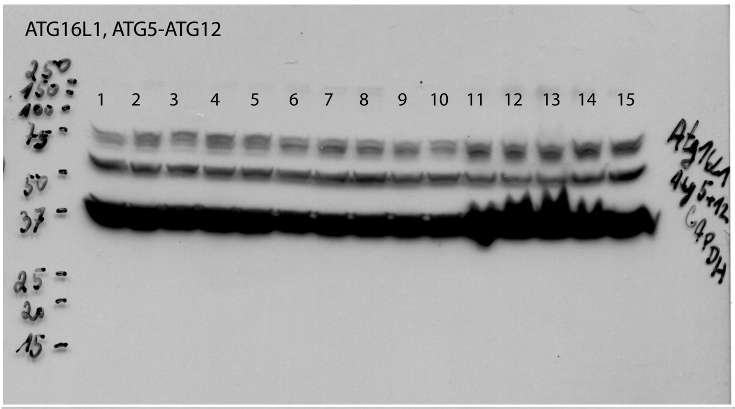

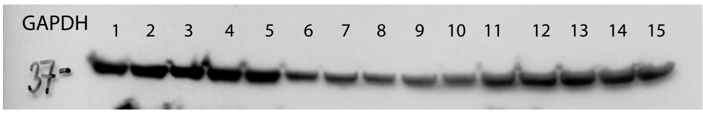

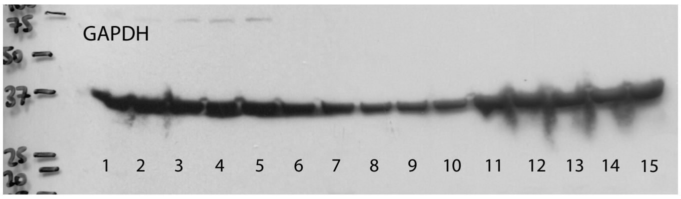

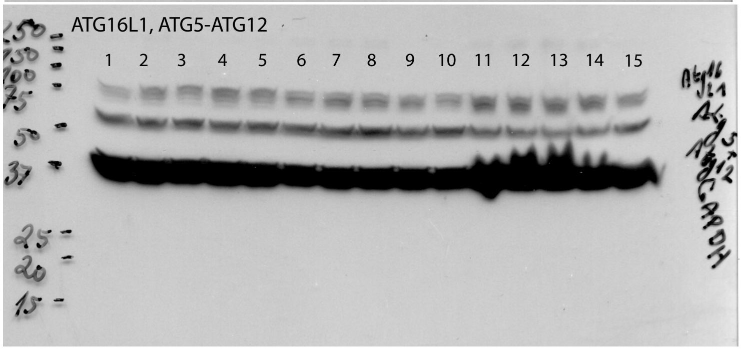

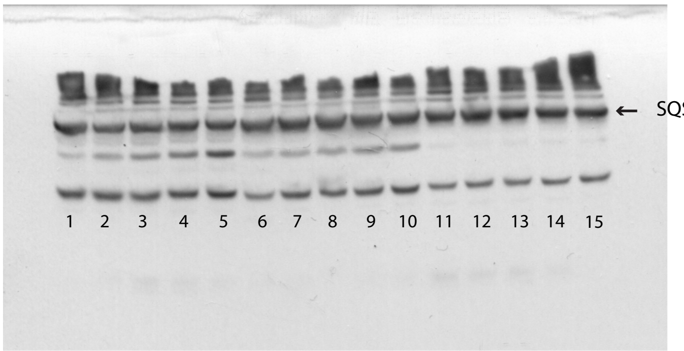

← SQSTM1/p62

1 MM.1S C
2 MM.1S WZ811 10 µM
3 MM.1S WZ811 20 µM
4 MM.1S WZ811 40 µM
5 MM.1S WZ811 80 µM
6 RPMI-S C
7 RPMI-S WZ811 10 µM
8 RPMI-S WZ811 20 µM
9 RPMI-S WZ811 40 µM
10 RPMI-S WZ811 80 µM
11 OPM-1 C
12 OPM-1 WZ811 10 µM
13 OPM-1 WZ811 20 µM
14 OPM-1 WZ811 40 µM
15 OPM-1 WZ811 80 µM

c-Myc, Mcl-1

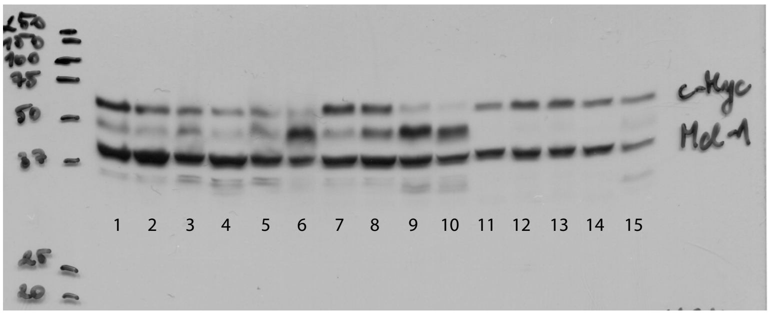

Mcl-1

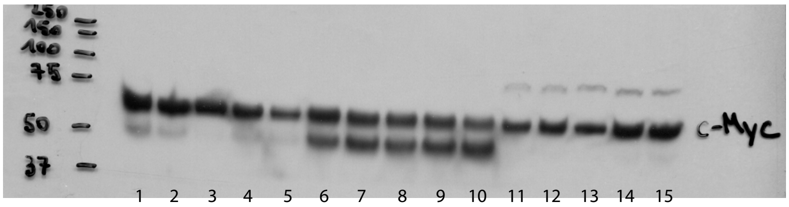

mTOR

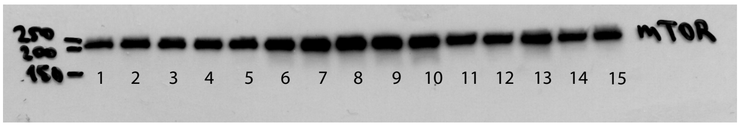

mTOR

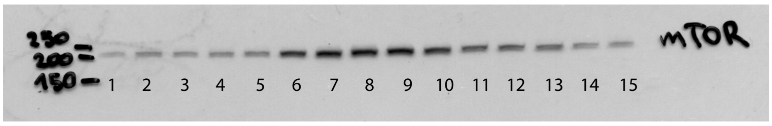

p-mTOR

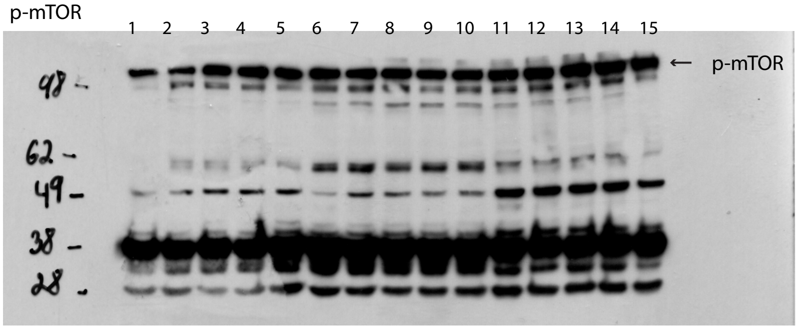

p-mTOR

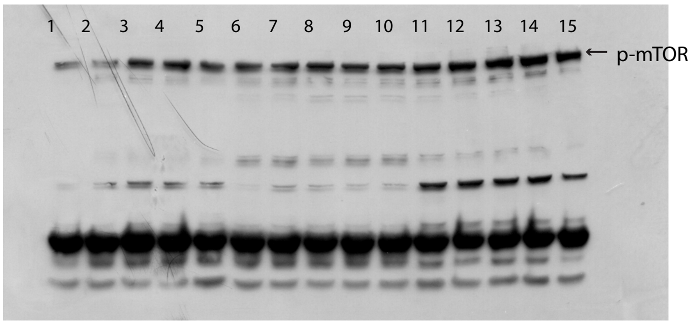

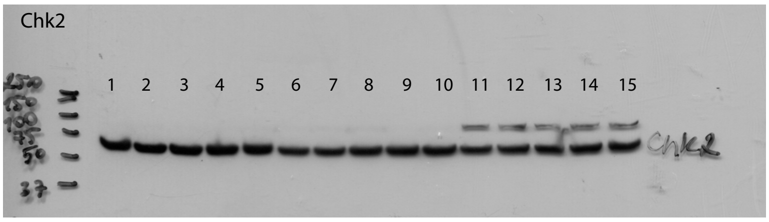

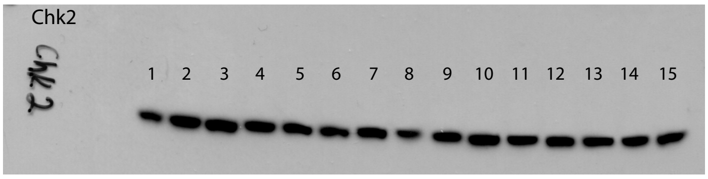

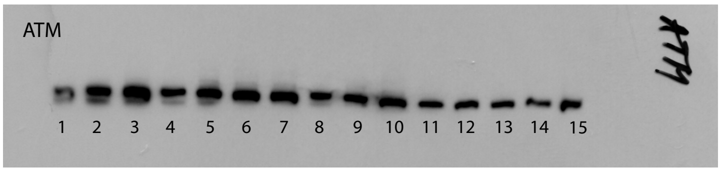

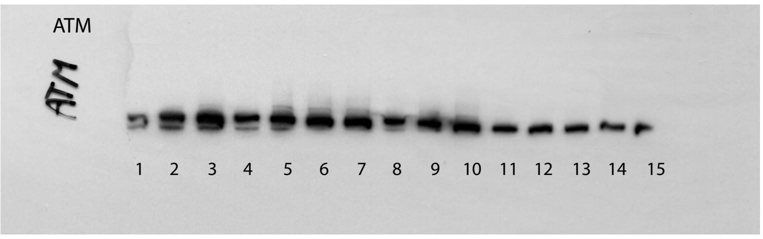

GAPDH

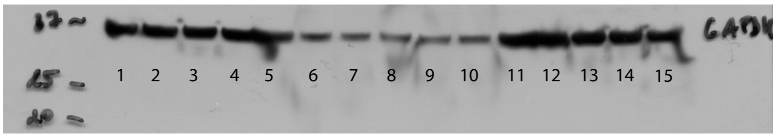

1 MM.1S C	6 RPMI-S C	11 OPM-1 C
2 MM.1S WZ811 10 μM	7 RPMI-S WZ811 10 μM	12 OPM-1 WZ811 10 μM
3 MM.1S WZ811 20 μM	8 RPMI-S WZ811 20 μM	13 OPM-1 WZ811 20 μM
4 MM.1S WZ811 40 μM	9 RPMI-S WZ811 40 μM	14 OPM-1 WZ811 40 μM
5 MM.1S WZ811 80 μM	10 RPMI-S WZ811 80 μM	15 OPM-1 WZ811 80 μM

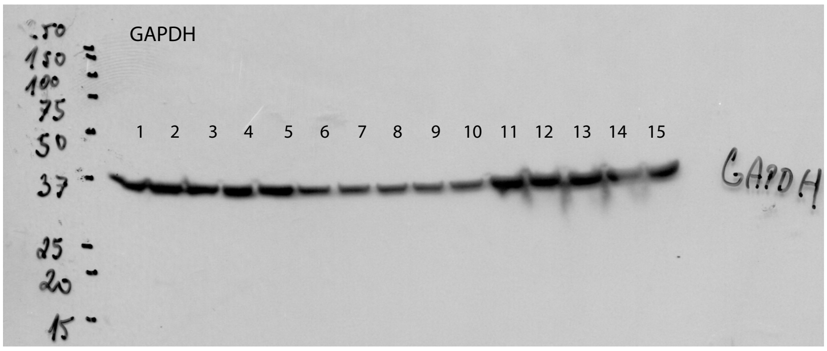

p-Cyclin B1

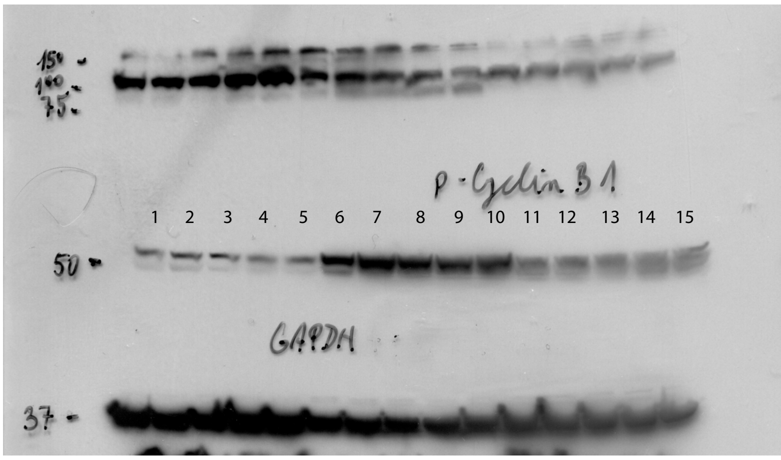

Cdc2, H2AX

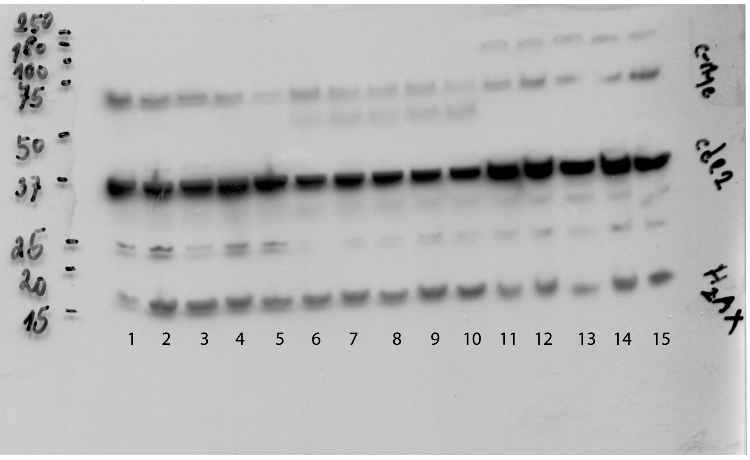

p-Cdc2, Cdc2

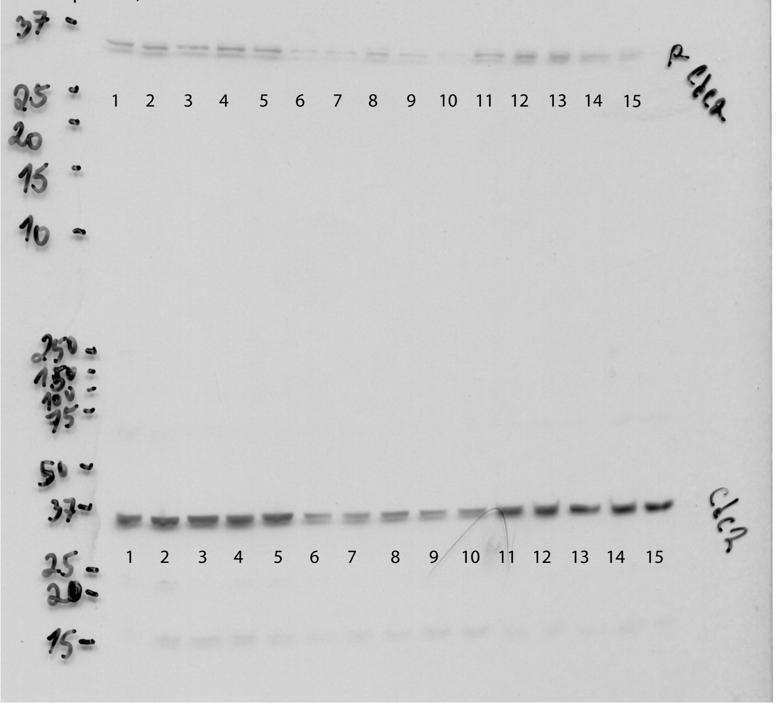

p-Cdc2

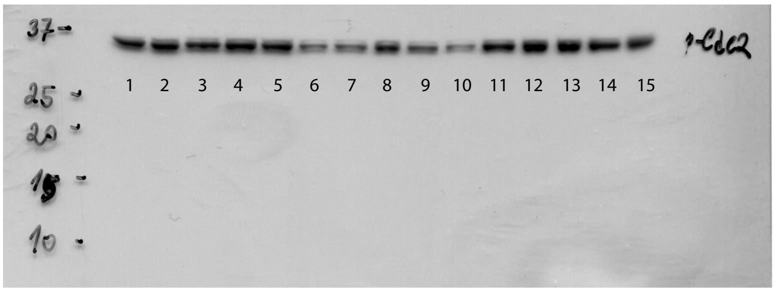

p-H2AX

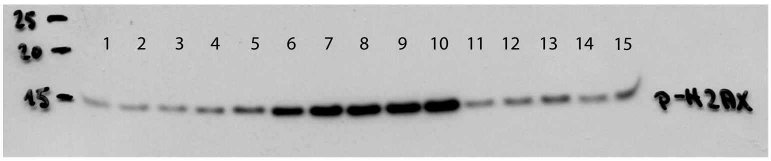

GAPDH

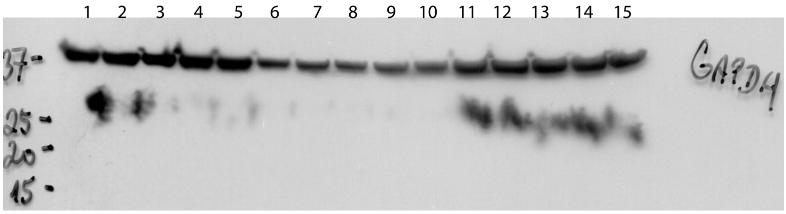

GAPDH

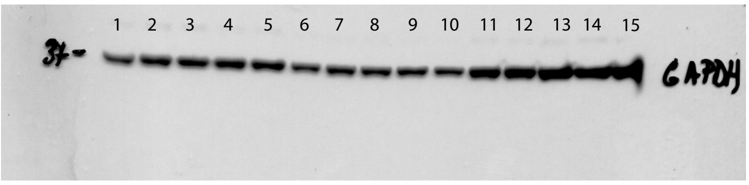

CXCR4

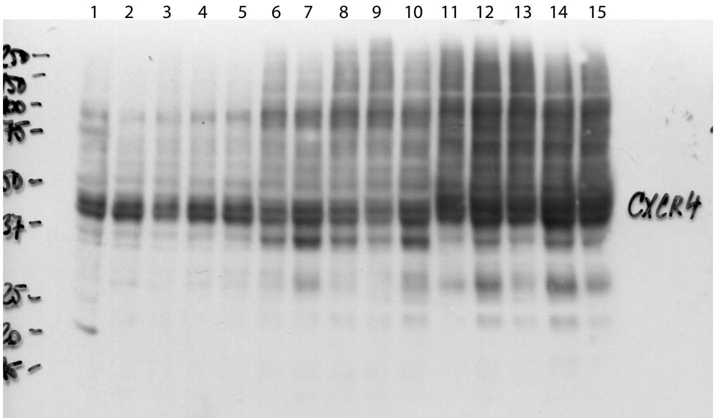

1 MM.1S C
2 MM.1S WZ811 10 μM
3 MM.1S WZ811 20 μM
4 MM.1S WZ811 40 μM
5 MM.1S WZ811 80 μM
6 RPMI-S C
7 RPMI-S WZ811 10 μM
8 RPMI-S WZ811 20 μM
9 RPMI-S WZ811 40 μM
10 RPMI-S WZ811 80 μM
11 OPM-1 C
12 OPM-1 WZ811 10 μM
13 OPM-1 WZ811 20 μM
14 OPM-1 WZ811 40 μM
15 OPM-1 WZ811 80 μM
