# Supplementary material for: Anti-myeloma activity of the CXCR4 antagonist WZ811
Source: J Mol Med (Berl). 2026 Feb 17;104(1):45. doi: 10.1007/s00109-026-02650-4 (PMC12913330; doi:10.1007/s00109-026-02650-4)
Supplement: Supplementary file 14 — (PDF 330 KB) [file 109_2026_2650_MOESM9_ESM.pdf]

CHARACTERISTICS	MM PATIENTS (N = 14)
Median age (range) - yr	62 (45 - 74)
Sex - no. (%)	
Female	6 (43)
Male	8 (57)
Stage of myeloma - no. (%)	
NDMM	6 (43)
RRMM	8 (57)
Medium serum creatinine (range) - µmol/l	79 (56.1 - 14)
Medium calcium (range) - mmol/l	2.3 (1.8 - 2.4)
Medium serum albumin (range) - g/l	38.5 (31.5 - 42)
Medium lactate dehydrogenase (range) - µkat/l	3 (2.7 - 3.6)
Hemoglobin (range) - g/l	133 (121 - 152)
Platelet count (range) - G/l	193.5 (84 - 290)
Type of myeloma (immunoglobulin)	
Median IgA (range) - mg/dl	4.8 (0.7 - 15.8)
No. of patients (%)	4 (28.6)
Median IgG (range) - mg/dl	6.4 (5.3 - 38.2)
No. of patients (%)	10 (71.4)
Type of light chain	
Median kappa (range) - g/l	1.4 (1.1 - 10.4)
No. of patients (%)	9 (64)
Median lambda (range) - g/l	1.2 (0.2 - .7)
No. of patients (%)	5 (36)
Median k/l (range)	1 (0.3 - 41.6)
Median bone marrow involvement (range) - (%)	19 (10 - 40)
Previous therapy of RRMM patients	
Median no. of previous treatment regimens (range)	1 (0 - 6)
1 - no.	2 (14)
2 - 3 - no.	3 (21)
≥ 4 - no.	3 (21)
Type of previous therapy - no. (%)	
Glucocorticoid	8 (57)
Proteasome inhibitor	7 (50)
Alkylator	3 (21)
Immunomodulator	7 (50)
Antracycline	2 (14)
Vinca alkaloids and derivatives	1 (7)
Other, including experimental therapy	2 (14)
Cytogenetic abnormalities - no. (%)	
t(11;14)	2 (14)
t(4;14)	0 (0)
t(14;16)	0 (0)
t(14;20)	1 (7)
Hyperdiploidy	5 (36)
del(17p13)/TP53 deletion	1 (7)
Gain(1q)/amp(1q21)	3 (21)
del(13q) or monosomy 13	2 (14)
MYC rearrangements	0 (0)
